# Supplementary material for: An algorithm for decoy-free false discovery rate estimation in XL-MS/MS proteomics
Source: Bioinformatics. 2024 Jun 28;40(Suppl 1):i428–36. doi: 10.1093/bioinformatics/btae233 (PMC11256928; doi:10.1093/bioinformatics/btae233)
Supplement: btae233_Supplementary_Data [file btae233_supplementary_data.zip › btae233_Supplementary_Data/Radivojac.246.marked.pdf]

Bioinformatics, 2024, 1–9
doi: DOI HERE
Advance Access Publication Date: Day Month Year
Paper

An algorithm for decoy-free false discovery rate estimation in XL-MS/MS proteomics

Yisu Peng,[1] Shantanu Jain[1,*] and Predrag Radivojac[1,*]

[1]Khoury College of Computer Sciences, Northeastern University, 177 Huntington Avenue, Boston, Massachusetts 02115, U.S.A.

*Corresponding authors: shajain@northeastern.edu, predrag@northeastern.edu

Abstract

Motivation: Cross-linking tandem mass spectrometry (XL-MS/MS) is an established analytical platform used to determine distance constraints between residues within a protein or from physically interacting proteins, thus improving our understanding of protein structure and function. To aid biological discovery with XL-MS/MS, it is essential that pairs of chemically linked peptides be accurately identified, a process that requires: (i) database search, that creates a ranked list of candidate peptide pairs for each experimental spectrum, and (ii) false discovery rate (FDR) estimation, that determines the probability of a false match in a group of top-ranked peptide pairs with scores above a given threshold. Currently, the only available FDR estimation mechanism in XL-MS/MS is the target-decoy approach (TDA). However, despite its simplicity, TDA has both theoretical and practical limitations that impact the estimation accuracy and increase run time over potential decoy-free approaches (DFAs).
Results: We introduce a novel decoy-free framework for FDR estimation in XL-MS/MS. Our approach relies on multi-sample mixtures of skew normal distributions, where the latent components correspond to the scores of correct peptide pairs (both peptides identified correctly), partially incorrect peptide pairs (one peptide identified correctly, the other incorrectly), and incorrect peptide pairs (both peptides identified incorrectly). To learn these components, we exploit the score distributions of first- and second-ranked peptide-spectrum matches (PSMs) for each experimental spectrum and subsequently estimate FDR using a novel expectation-maximization algorithm with constraints. We evaluate the method on ten datasets and provide evidence that the proposed DFA is theoretically sound and a viable alternative to TDA owing to its good performance in terms of accuracy, variance of estimation, and run time.
Availability: https://github.com/shawn-peng/xlms

1. Introduction

Cross-linking mass spectrometry (XL-MS) proteomics has emerged as a key technique in molecular and structural biology, particularly for an exploration of protein assemblies and protein-protein interactions under native cellular conditions (Sinz, 2003, 2006; Yu and Huang, 2018; Piersimoni *et al.*, 2021). In a typical experiment, a chemical reagent (linker) capable of forming covalent bonds with side-chains of specific residues (e.g., lysine) on each end, is first introduced to the protein mixture. The sample is then digested and processed using liquid chromatography (LC) coupled with tandem mass spectrometry (MS/MS) to identify pairs of inter- or intra-protein cross-linked peptides. Since the chemically linked residues must be located within the distance of the spacer arm of the linker (e.g., 10-30Å), the experiment provides a set of distance constraints that can be key to resolving protein structure and interaction sites, often in combination with other techniques (Rappsilber, 2011; Piersimoni *et al.*, 2021). However, XL-MS also offers distinct advantages including the rapid interrogation of protein isoforms, post-translationally modified proteins, membrane proteins, and disordered proteins (Piersimoni *et al.*, 2021). The digestion step in XL-MS/MS further removes restrictions on the protein size, localization or conformational landscape, although the dynamic range of modern analytical instrumentation still limits the studies to relatively abundant proteoforms.

Despite its promise, XL-MS/MS has not fully matured and a number of challenges remain (Piersimoni *et al.*, 2021). One key challenge is related to the data processing pipelines, particularly the computational and statistical difficulties associated with identification and quantification of cross-linked peptides (Yang *et al.*, 2012). XL-MS/MS peptide identification follows a similar workflow to the traditional MS/MS (Steen and Mann, 2004). The first step is database search (Yates *et al.*, 1995; Perkins *et al.*, 1999;

© The Author 2024. Published by Oxford University Press. All rights reserved. For permissions, please e-mail: journals.permissions@oup.com

Kim and Pevzner, 2014; Kong *et al.*, 2017), where the experimental spectra from the instrument are searched against a database of theoretical spectra of peptides (peptide pairs in XL-MS/MS) whose mass (sum of masses, including the linker), is within the instrument's tolerance of the measured mass. This produces a ranked list of peptides (peptide pairs) for each experimental spectrum, or peptide-spectrum matches (PSMs), with only the top-ranked PSM eligible for downstream identification if its score is sufficiently large. The second step is a procedure devised to control the error rate of identifications (Storey, 2002; Choi and Nesvizhskii, 2008; Aggarwal and Yadav, 2016; Burger, 2018), with the objective to determine the threshold above which all top PSMs will be considered identified with the false discovery rate (FDR) below a predetermined value; e.g., FDR = 1%.

In XL-MS/MS, both steps add complexity to the traditional pipeline. For example, an XL-MS/MS search engine must scan a database of peptide pairs instead of single peptides (Rinner *et al.*, 2008; Hoopmann *et al.*, 2015; Ji *et al.*, 2016; Netz *et al.*, 2020), thus increasing the run time (quadratically) and the competition for each experimental spectrum. Similarly, FDR control is more difficult in part because the incorrect identifications include PSMs where both peptides in a pair are incorrectly identified and also PSMs where one peptide is correctly identified and the other is not (Walzthoeni *et al.*, 2012). Depending on the fragmentation patterns and the search engine, these so-called partially incorrect identifications can have relatively high scores. Overall, the increased search space and the complexity of error control both contribute to the smaller fraction of identified spectra compared to the traditional MS/MS, for the same estimated FDR (Piersimoni *et al.*, 2021).

To control for FDR, traditional MS/MS platforms rely on both target-decoy (TDA) and decoy-free (DFA) approaches, with TDAs being the preferred option. TDAs typically search a database of peptides that are potentially present in the sample (target sequences) together with an equal-sized set of peptides that cannot be present in the sample (decoy sequences; often reversed target sequences). The high-scoring decoy PSMs are then used to estimate the number of false target identifications (Elias and Gygi, 2007; Jeong *et al.*, 2012). In contrast, DFAs typically fit two-component mixture models to the distribution of top PSM scores, where one of the latent components corresponds to the correct and the other to incorrect identifications. The expectation-maximization (EM) algorithm is then used to resolve the component distributions using parametric families such as Gaussian, gamma or skew normal (Keller *et al.*, 2002; Li, 2008; Peng *et al.*, 2020). In contrast, TDA is an exclusive error control mechanism in XL-MS/MS (Walzthoeni *et al.*, 2012) and the absence of DFAs may be due to the fact that the set of top-ranked PSM scores cannot be easily modeled using parametric two-component mixtures, especially the heterogeneous distribution of incorrect PSMs.

Despite their simplicity and prevalence in a standard workflow, TDAs exhibit important theoretical and practical limitations (Kall *et al.*, 2008a,b; Gupta *et al.*, 2011; Cooper, 2011, 2012; He *et al.*, 2015; Danilova *et al.*, 2019; Peng *et al.*, 2020). Theoretically, FDR estimates can be greater than 1, and likewise, the strategy of competing target with decoy peptides for the available experimental spectra is problematic and may lead to biased estimates. Practically, the search time for TDA is increased, it cannot be applied to de novo searches (Dancik *et al.*, 1999; Frank and Pevzner, 2005), it shows high-variance of the score cutoffs at low FDR (Peng *et al.*, 2020), and the estimation is inaccurate for the samples with low amounts of biological material (Li *et al.*, 2015; Budnik *et al.*, 2018; Peng *et al.*, 2020). The problems with run time are further amplified in XL-MS/MS, quadrupling the search times over those that could be achieved with DFAs.

To address these problems, this study introduces a novel DFA for FDR estimation in XL-MS/MS. We exploit the score distributions of top-ranked and second-ranked PSMs, and model them as five-component mixtures (with shared parameters) from the skew normal family. We then devise a multi-sample EM algorithm with constraints to resolve the latent components. Our results show that this method holds promise for enhancing the accuracy and reliability of XL-MS/MS studies and is an attractive alternative to TDAs.

2. Background

2.1. Terminology and Notation

Let $\mathcal{X} = \{x_i\}$ be a set of spectra collected from a mass spectrometer and $\mathcal{P} = \{(p_\alpha, p_\beta)_j\}$ a set of candidate pairs of (sorted) peptides. A search engine produces a set of triplets $(x, (p_\alpha, p_\beta), s) \in \mathcal{X} \times \mathcal{P} \times \mathbb{R}$, where s is the score assigned to the PSM $(x, (p_\alpha, p_\beta))$. The higher the score, the more likely that the spectrum x was generated from (p_α, p_β).

Let now x be generated from an unknown peptide pair (q_α, q_β) and let $((x, (p_\alpha, p_\beta)_1, s_1), (x, (p_\alpha, p_\beta)_2, s_2), \ldots)$ be a ranked list of PSMs from a search engine for x such that $s_1 \geq s_2 \geq$ and so on. A PSM $(x, (p_\alpha, p_\beta))$ for which $(p_\alpha, p_\beta) = (q_\alpha, q_\beta)$ is called the *correct match*. If only one of the peptides matches the ground truth we refer to these as *partially incorrect matches*, whereas all other PSMs involving x are called *incorrect matches*. Furthermore, given the ranked list of PSMs, the PSM with the highest score, $(x, (p_\alpha, p_\beta)_1)$, is called the top-ranked or first PSM, $(x, (p_\alpha, p_\beta)_2)$ is called the the second-ranked or second PSM, etc. We similarly distinguish among partially incorrect and incorrect PSMs.

An MS/MS analysis pipeline looks at top-ranked PSMs and determines a threshold τ such that the pair of peptides (p_α, p_β) from each top hit $(x, (p_\alpha, p_\beta))$ is considered *identified* when the score s from $(x, (p_\alpha, p_\beta), s)$ satisfies $s \geq \tau$. If, further, $(p_\alpha, p_\beta) = (q_\alpha, q_\beta)$, it is considered to be the *correct identification*. The threshold τ must be established to satisfy a desired estimated FDR for the biological study.

2.2. Skew Normal Distributions

Azzalini (1985) introduced the skew normal (SN) family of distributions as a generalization of the normal family that allows for skewness. It has a location (μ), a scale (σ), and a shape (λ) parameter, where λ controls for skewness. The distribution is right skewed when $\lambda > 0$, left skewed when $\lambda < 0$, and reduces to a normal distribution when $\lambda = 0$. The probability density function (pdf) of a random variable $S \sim \mathrm{SN}(\mu, \sigma, \lambda)$ is given by

$$f(s; \mu, \sigma, \lambda) = \frac{2}{\sigma} \phi\left(\frac{s-\mu}{\sigma}\right) \Phi\left(\frac{\lambda(s-\mu)}{\sigma}\right), \; s \in \mathbb{R},$$

where $\mu, \lambda \in \mathbb{R}$, $\sigma \in \mathbb{R}^+$, ϕ and Φ are the pdf and the cumulative distribution function (cdf) of $\mathrm{N}(0, 1)$, respectively. Alternatively, SN family can be parameterized by Δ and Γ (Table 1), instead of λ and σ. The alternate parametrization naturally arises in the following stochastic representation of a SN random variable (Henze, 1986)

Table 1. Relationship between the alternate and canonical SN parameters.

Alternate Parametrization		Related Quantities
canonical → alternate	alternate → canonical	
$\Delta = \sigma\delta$<br>$\Gamma = \sigma^2 - \Delta^2$	$\lambda = \text{sign}(\Delta)\sqrt{\Delta^2/\Gamma}$<br>$\sigma = \sqrt{\Gamma + \Delta^2}$	$\delta = \frac{\lambda}{\sqrt{1+\lambda^2}}$

$$S \sim \text{SN}(\mu, \sigma, \lambda) \Rightarrow S \overset{d}{=} \mu + \Delta T + \Gamma^{1/2} U, \tag{1}$$

where $T \sim \text{TN}_+(0, 1)$, the standard normal distribution truncated below 0; $U \sim \text{N}(0, 1)$; $\overset{d}{=}$ reads as "equal in distribution". The stochastic representation is exploited in developing expectation-maximization (EM) algorithms for maximum likelihood estimation of SN distributions and their mixtures (Lin *et al.*, 2007; Lin, 2009).

2.3. Target-Decoy FDR Estimation

In a TDA, the spectra $\mathcal{X}$ are searched against a concatenated database of target and decoy sequences. To estimate the FDR at a threshold τ, Walzthoeni *et al.* (2012) derived the following expression

$$\text{FDR}(\tau) \overset{est}{=} \frac{\text{TD}(\tau) - \text{DD}(\tau)}{\text{TT}(\tau)},$$

where $\text{TT}(\tau)$ is the number of (top) PSMs matched to target sequences for both peptides, $\text{TD}(\tau)$ is the number of PSMs with exactly one peptide matched to decoy sequences, and $\text{DD}(\tau)$ is number of PSMs with both peptides matched to decoy sequences.

3. Data and Database Search

We downloaded ten XL-MS/MS datasets from the PRoteomics IDEntifications (PRIDE) database (Perez-Riverol *et al.*, 2022), as shown in Table 2. The data were downloaded as raw spectra. We used ProteoWizard to convert the raw spectra into peaks files in the .mzML format. After the peaks were identified, we used OpenPepXL (Netz *et al.*, 2020) to run a search for each file against the protein sequence database as described in the original paper. For each file, we ran two searches, one with decoy sequences for TDA experiments and another without decoy sequences for DFA experiments. To run a TDA search, we concatenated the original database with reversed protein sequences. The search parameters for each dataset were also identical to those in the original papers.

After the search was completed, we extracted the scores for the top two PSMs corresponding to each experimental spectrum. If a spectrum only matched to one candidate peptide pair, we marked the second score as missing. If the top two candidates for one spectrum had the same peptides but differed on the position of cross-linked residues, we retained only the top one and promoted the third scoring peptide pair, if available, to the second position.

4. Methods

4.1. Approach

To estimate the FDR in an XL-MS/MS search, we build on our mixture approach for the traditional LC-MS/MS search (Peng *et al.*, 2020). The proposed method uses a mixture of skew normal distributions to model the PSM scores not only for the top hits but also for the second hits to improve the estimation of the latent components.

Let S_1 and S_2 be random variables giving the top and second PSM scores for a spectrum, respectively, where S_1 and S_2 could be coming from a correct match (the two chains matched to the correct peptide pair), partially incorrect match (only one chain matched to the correct peptide) and an incorrect match (both chains matched to incorrect peptides). We define C as the random variable giving the score corresponding to the correct match; J_1 and J_2 as those for the highest- and second highest-scoring partially incorrect matches, respectively; I_1 and I_2 as those for highest- and second highest-scoring incorrect matches, respectively. Note that S_1 and S_2 are observed in the data, whereas C, J_1, J_2, I_1 and I_2 are not observed (latent). Our approach for FDR estimation relies on modeling S_1 and S_2 as mixtures of C, J_1, J_2, I_1 and I_2 as latent components, and fitting the data to uncover their distributions. As justified by our experimental results, incorporating the second score has advantages over a model based on the top score only. We model each latent variable using a skew normal distribution; i.e., $C \sim \text{SN}(\theta_\text{C})$, $J_1 \sim \text{SN}(\theta_{\text{J}_1})$, $J_2 \sim \text{SN}(\theta_{\text{J}_2})$, $I_1 \sim \text{SN}(\theta_{\text{I}_1})$ and $I_2 \sim \text{SN}(\theta_{\text{I}_2})$, where $\theta_\text{Y} = (\mu_\text{Y}, \sigma_\text{Y}, \lambda_\text{Y})$ contains the SN parameters, for $\text{Y} \in \mathbf{Y} = \{\text{C}, \text{J}_1, \text{J}_2, \text{I}_1, \text{I}_2\}$.

We next describe our model and estimation algorithm focusing on the approach that uses both top- and second-ranked PSMs. In Section 5, however, we evaluate this algorithm against a one-sample model that only relies on the top-ranked PSMs and TDA.

4.2. Two-Sample Statistical Model

We model S_1 as a mixture distribution of $\text{SN}(\theta_\text{C})$, $\text{SN}(\theta_{\text{J}_1})$ and $\text{SN}(\theta_{\text{I}_1})$ as components, since the top score can only come from the correct match (C), top-scoring partially incorrect match (J_1) or the top-scoring incorrect match (I_1). Now, S_2 may also come from

Name	PRIDE ID	Organism	Cross-linker	Precursor tolerance	Fragment tolerance	Number of spectra
ALott	PXD032037	*H. sapiens*	DSS	10 ppm	20 ppm	505791
Alinden	PXD031985	*H. sapiens*	BS3	20 ppm	20 ppm	301826
CPSF	PXD031242	*H. sapiens*	DSS	10 ppm	20 ppm	198385
D1810	PXD013470	*A. thaliana*	DSS	10 ppm	20 ppm	360259
MS2000225	PXD022119	*H. sapiens*	BS3	10 ppm	20 ppm	53918
QE	PXD014738	*C. thermophilum*	DSS	10 ppm	50 ppm	187039
RPA	PXD028637	*S. cerevisiae*	BS3	5 ppm	5 ppm	8826
Alban	PXD033409	*H. sapiens*	DSS	10 ppm	20 ppm	31659
Ecoli	PXD003381	*E. coli*	DEST	5 ppm	5 ppm	277748
Peplib	PXD014337	*S. pyogenes*	DSS	10 ppm	20 ppm	98070

Table 2. Summary of datasets and search parameters used in this study. All datasets are available from PRIDE (Perez-Riverol *et al.*, 2022).

C (when $S_1 \neq C$), J_1 (when $S_1 \neq J_1$) and I_1 (when $S_1 \neq I_1$). However, it can alternatively come from J_2 (when $S_1 = J_1$ and J_2 is greater than C, I_1 and I_2) or I_2 (when $S_1 = I_1$ and I_2 is greater than C, J_1 and J_2). Hence, we model it as a mixture of $\mathrm{SN}(\theta_{\mathrm{C}})$, $\mathrm{SN}(\theta_{\mathrm{J}_1})$, $\mathrm{SN}(\theta_{\mathrm{J}_2})$, $\mathrm{SN}(\theta_{\mathrm{I}_1})$ and $\mathrm{SN}(\theta_{\mathrm{I}_2})$. Formally,

$$S_1 \sim w_{\mathrm{C}}\mathrm{SN}(\theta_{\mathrm{C}}) + w_{\mathrm{J}}\mathrm{SN}(\theta_{\mathrm{J}_1}) + w_{\mathrm{I}_1}\mathrm{SN}(\theta_{\mathrm{I}_1}),$$
$$S_2 \sim v_{\mathrm{C}}\mathrm{SN}(\theta_{\mathrm{C}}) + v_{\mathrm{J}_2}\mathrm{SN}(\theta_{\mathrm{J}_1}) + v_{\mathrm{J}_2}\mathrm{SN}(\theta_{\mathrm{J}_2}) + v_{\mathrm{I}_1}\mathrm{SN}(\theta_{\mathrm{I}_1}) + v_{\mathrm{I}_2}\mathrm{SN}(\theta_{\mathrm{I}_2}),$$

where $w_{\mathrm{X}} > 0$ and $\sum_{\mathrm{X}} w_{\mathrm{X}} = 1$ for $\mathrm{X} \in \{\mathrm{C}, \mathrm{J}_1, \mathrm{I}_1\}$ and $v_{\mathrm{X}} > 0$ and $\sum_{\mathrm{X}} v_{\mathrm{X}} = 1$ for $\mathrm{X} \in \{\mathrm{C}, \mathrm{J}_1, \mathrm{J}_2, \mathrm{I}_1, \mathrm{I}_2\}$ give the mixing proportions (weights) of the components within the mixtures. The sharing of $\mathrm{SN}(\theta_{\mathrm{C}})$, $\mathrm{SN}(\theta_{\mathrm{J}_1})$ and $\mathrm{SN}(\theta_{\mathrm{I}_1})$ between the two mixtures allows incorporating information from both scores to learn the parameters θ_{C}, θ_{J_1} and θ_{I_1}. The additional components, $\mathrm{SN}(\theta_{\mathrm{J}_2})$ and $\mathrm{SN}(\theta_{\mathrm{I}_2})$, in the second mixture and the differing mixing proportions allow capturing the distributional differences between S_1 and S_2.

4.3. Constraints

In addition to the mixture formulation with parameter sharing, we incorporate inequality constraints on the mixing proportions (weights) that naturally emerge due to the latent structure between the top two scores. Additionally, we incorporate intuitive constraints between the density functions of the latent variables.

4.3.1. Weight Constraints

Due to the nature of the relationship between the top two scores from the same spectra, they cannot come from the same component. Furthermore, the second score can be J_2 or I_2 only if the top score comes from J_1 or I_1, respectively. These observations lead to several inequality constraints between the mixing proportions. For example, when the top score comes from C, the second score has to come from I_1 or J_1 and cannot be I_2 or J_2 because they are lower than I_1 and J_1, respectively. This implies that that the proportion of C in the top score is upper bounded by the sum of the proportions of J_1 and I_1 in the second score; i.e., $w_{\mathrm{C}} \leq v_{\mathrm{I}_1} + v_{\mathrm{J}_1}$. The constraint set A below was derived in this manner.

Constraint set A	Constraint set B
$w_{\mathrm{C}} \leq v_{\mathrm{J}_1} + v_{\mathrm{I}_1}$	$w_{\mathrm{C}} \leq v'_{\mathrm{J}_1} + v'_{\mathrm{I}_1} + v_{\Phi}$
$w_{\mathrm{J}_1} \leq v_{\mathrm{C}} + v_{\mathrm{J}_2} + v_{\mathrm{I}_1}$	$w_{\mathrm{J}_1} \leq v'_{\mathrm{C}} + v'_{\mathrm{J}_2} + v'_{\mathrm{I}_1} + v_{\Phi}$
$w_{\mathrm{I}_1} \leq v_{\mathrm{C}} + v_{\mathrm{J}_1} + v_{\mathrm{I}_2}$	$w_{\mathrm{I}_1} \leq v'_{\mathrm{C}} + v'_{\mathrm{J}_1} + v'_{\mathrm{I}_2} + v_{\Phi}$
$v_{\mathrm{C}} \leq w_{\mathrm{J}_1} + w_{\mathrm{I}_1}$	$v'_{\mathrm{C}} \leq w_{\mathrm{J}_1} + w_{\mathrm{I}_1}$
$v_{\mathrm{J}_1} \leq w_{\mathrm{C}} + w_{\mathrm{I}_1}$	$v'_{\mathrm{J}_1} \leq w_{\mathrm{C}} + w_{\mathrm{I}_1}$
$v_{\mathrm{I}_1} \leq w_{\mathrm{C}} + w_{\mathrm{J}_1}$	$v'_{\mathrm{I}_1} \leq w_{\mathrm{C}} + w_{\mathrm{J}_1}$
$v_{\mathrm{J}_2} \leq w_{\mathrm{J}_1}$	$v'_{\mathrm{J}_2} \leq w_{\mathrm{J}_1}$
$v_{\mathrm{I}_2} \leq w_{\mathrm{I}_1}$	$v'_{\mathrm{I}_2} \leq w_{\mathrm{I}_1}$, $\quad (v'_{\mathrm{Y}} = (1 - v_{\Phi})v_{\mathrm{Y}})$

In our implementation, we modify the constraint set A to account for the cases where the search gives only a single match for a spectrum; i.e., when the second score is missing. We however observed that the second scores are not missing at random. Missing second scores occur preferentially at the left tail of the top-score distribution; see Supplementary Materials. Due to non-random missingness of the second score, the constraint set A becomes invalid. To address this issue, we derive the constraint set B which gives a set of valid constraints, irrespective of the non-random nature of the missing second scores (derivation in Supplementary Materials). The constraints explicitly incorporate the proportion of the spectra with missing second scores, v_{Φ}, as a constant. All second-score mixing proportions are scaled and included as $v'_{\mathrm{Y}} = (1 - v_{\Phi})v_{\mathrm{Y}}, \forall \mathrm{Y} \in \mathbf{Y}$, for brevity. We will incorporate the constraint set B in our algorithm.

4.3.2. Density constraints

In addition to the constraints between mixing proportions, we also enforced constraints between the density functions of the components in a pairwise manner. Since the correct scores have higher values than partially incorrect scores on average, we expect the mode of the C density to be higher than that of J_1. We similarly expect C (J_1) to have a higher density at any given point in its right (left) tail compared to J_1 (C); we also expect such relationships between other pairs of densities such as C and I_1, J_1 and I_1, J_1 and J_2 and I_1 and I_2. We formalize such constraints between a pair of densities, f and g, using a strict partial order $f \succ g$ (f dominates g) defined by the following constraints

$$\mathrm{mode}(f) > \mathrm{mode}(g)$$
$$f(x) > g(x), \quad \forall x > \mathrm{mode}(f)$$
$$g(x) > f(x), \quad \forall x < \mathrm{mode}(g),$$

where $\mathrm{mode}(f) = \mathrm{argmax}_x f(x)$ is the mode of density f. We enforce the following pairwise constraints in our approach

$$f_{\mathrm{C}} \succ f_{\mathrm{J}_1}, \quad f_{\mathrm{J}_1} \succ f_{\mathrm{J}_2}, \quad f_{\mathrm{J}_2} \succ f_{\mathrm{I}_1}, \quad f_{\mathrm{I}_1} \succ f_{\mathrm{I}_2}.$$

Due to transitivity of the strict partial order, the following ordering of the densities holds: $f_{\mathrm{C}} \succ f_{\mathrm{J}_1} \succ f_{\mathrm{J}_2} \succ f_{\mathrm{I}_1} \succ f_{\mathrm{I}_2}$.

4.4. Algorithm

We derive an expectation-maximization (EM) algorithm-based maximum likelihood estimation with several weight and density constraints to capture the structure inherent to XL-MS/MS data. To enforce the weight constraints we convert the so-called $\mathcal{Q}$-function in the maximization step of the EM algorithm into a Lagrangian function with additional terms and parameters for the constraints. The density constraints are enforced in each step by performing a binary search between the old and the new parameters.

4.4.1. Derivation of the $\mathcal{Q}$-function

We first write the log-likelihood function for the model as

$$\mathcal{L}(\zeta) = \sum_{s_1 \in \mathbb{S}_1} \log\left(\sum_{\mathrm{X} \in \mathbf{X}} w_{\mathrm{X}} f_{\mathrm{SN}}(s_1; \theta_{\mathrm{X}})\right) + \sum_{s_2 \in \mathbb{S}_2} \log\left(\sum_{\mathrm{Y} \in \mathbf{Y}} v_{\mathrm{Y}} f_{\mathrm{SN}}(s_2; \theta_{\mathrm{Y}})\right), \tag{2}$$

where $\mathbf{X} = \{\mathrm{C}, \mathrm{J}_1, \mathrm{I}_1\}$ and $\mathbf{Y} = \{\mathrm{C}, \mathrm{J}_1, \mathrm{J}_2, \mathrm{I}_1, \mathrm{I}_2\}$. Variable $\boldsymbol{\zeta} = \{\{w_{\mathrm{X}}\}_{\mathrm{X} \in \mathbf{X}}, \{v_{\mathrm{Y}}\}_{\mathrm{Y} \in \mathbf{Y}}, \{\theta_{\mathrm{Y}}\}_{\mathrm{Y} \in \mathbf{Y}}\}$ contains all model parameters. Next, we introduce the hidden variables for the EM framework. Let $\{W_{\mathrm{X}}(s_1)\}_{\mathrm{X} \in \mathbf{X}}$ and $\{V_{\mathrm{Y}}(s_2)\}_{\mathrm{Y} \in \mathbf{Y}}$ be two sets of binary variables giving the source component for s_1 and s_2, respectively. If s_1

(s_2) comes from component X (Y), $W_{\mathrm{X}}(s_1) = 1$ ($V_{\mathrm{Y}}(s_2) = 1$), otherwise $W_{\mathrm{X}}(s_1) = 0$ ($V_{\mathrm{Y}}(s_2) = 0$). Each score, given its component affiliation, is a SN variable and consequently has an associated $\mathrm{TN}_+(0,1)$ variable from its stochastic representation (Section 2.2), one for each component it may come from. Let $\{T_{\mathrm{X}}(s_1)\}_{\mathrm{X}\in\mathbf{X}}$ and $\{T_{\mathrm{Y}}(s_2)\}_{\mathrm{Y}\in\mathbf{Y}}$ be the set of such $\mathrm{TN}_+(0,1)$ variables for s_1 and s_2, respectively. Omitting s_1 and s_2 as arguments of $W_{\mathrm{X}}(s_1), V_{\mathrm{Y}}(s_2), T_{\mathrm{X}}(s_1)$ and $T_{\mathrm{Y}}(s_2)$, the complete data log-likelihood up to an additive constant in ζ is given by

$$\mathcal{L}_{\mathrm{cmp}}(\zeta) = \sum_{s_1\in\mathbb{S}_1}\sum_{\mathrm{X}\in\mathbf{X}} W_{\mathrm{X}}\left(\log w_{\mathrm{X}} - \frac{q(s_1, T_{\mathrm{X}}, T_{\mathrm{X}}^2, \theta_{\mathrm{X}})}{2}\right) + \sum_{s_2\in\mathbb{S}_2}\sum_{\mathrm{Y}\in\mathbf{Y}} V_{\mathrm{Y}}\left(\log v_{\mathrm{Y}} - \frac{q(s_2, T_{\mathrm{Y}}, T_{\mathrm{Y}}^2, \theta_{\mathrm{Y}})}{2}\right),$$

where $q(s,t,\tau,\theta) = \log\Gamma + \frac{(s-\mu)^2 - 2(s-\mu)\Delta t + (\Delta^2+\Gamma)\tau}{\Gamma}$. The $\mathcal{Q}$-function for the EM algorithm is defined as the conditional expectation of $\mathcal{L}_{\mathrm{cmp}}(\zeta)$ given the observed data $(\mathbb{S}_1, \mathbb{S}_2)$, computed using the current estimate of the parameters, $\bar{\zeta}$. The hidden variables $\{W_{\mathrm{X}}(s_1)\}_{\mathrm{X}\in\mathbf{X}}$, $\{V_{\mathrm{Y}}(s_2)\}_{\mathrm{Y}\in\mathbf{Y}}$, $\{T_{\mathrm{X}}(s_1)\}_{\mathrm{X}\in\mathbf{X}}$ and $\{T_{\mathrm{Y}}(s_2)\}_{\mathrm{Y}\in\mathbf{Y}}$ are the random quantities in $\mathcal{L}_{\mathrm{cmp}}(\zeta)$. Thus the expectation is taken with respect to their conditional distribution given $\mathbb{S}_1$ and $\mathbb{S}_2$. The current parameters, $\bar{\zeta}$, are only used for taking the expectation and they do not replace the parameters in the expression for $\mathcal{L}_{\mathrm{cmp}}(\zeta)$. Consequently, the $\mathcal{Q}$-function is a function of both ζ and $\bar{\zeta}$. It is given by

$$\mathcal{Q}(\zeta|\bar{\zeta}) = \sum_{s_1\in\mathbb{S}_1}\sum_{\mathrm{X}\in\mathbf{X}} \bar{\omega}_{\mathrm{X}}(s_1)\left(\log w_{\mathrm{X}} - \frac{Q(s_1, \theta_{\mathrm{X}}, \bar{\theta}_{\mathrm{X}})}{2}\right) + \sum_{s_2\in\mathbb{S}_2}\sum_{\mathrm{Y}\in\mathbf{Y}} \bar{\nu}_{\mathrm{Y}}(s_2)\left(\log v_{\mathrm{Y}} - \frac{Q(s_2, \theta_{\mathrm{Y}}, \bar{\theta}_{\mathrm{Y}})}{2}\right),$$

where $Q(s,\theta,\bar{\theta}) = q(s, \xi_1(s,\bar{\theta}), \xi_2(s,\bar{\theta}), \theta)$; $\xi_1(s,\theta)$ and $\xi_2(s,\theta)$ are the first and second moments of a truncated normal distribution, respectively, as defined in Table 3; $\bar{\omega}_{\mathrm{X}}(s_1)$ ($\bar{\nu}_{\mathrm{X}}(s_2)$) is the probability that s_1 (s_2) comes from X (Y) under the current parameters (Table 3). The EM approach relies on finding new parameters, $\ddot{\zeta}$, at each iteration, that increase the $\mathcal{Q}$-function; i.e., $\mathcal{Q}(\ddot{\zeta}|\bar{\zeta}) \geq \mathcal{Q}(\bar{\zeta}|\bar{\zeta})$, to indirectly increase the log-likelihood (Eq. 2).

4.4.2. Component parameter updates

To update the component parameters $\{\theta_{\mathrm{Y}}\}_{\mathrm{Y}\in\mathbf{Y}}$, we adopt the Expectation Conditional Maximization (ECM) approach of optimizing $\mathcal{Q}(\zeta|\bar{\zeta})$ one parameter at a time, as it leads to simpler closed-form update equations without compromising the monotonicity of the $\mathcal{Q}$-function and the log-likelihood (Meng and Rubin, 1993); see Supplementary Materials. Taking the partial derivative of $\mathcal{Q}(\zeta|\bar{\zeta})$ with respect to $\mu_{\mathrm{Y}}, \Delta_{\mathrm{Y}}$ and Γ_{Y} and equating them to 0, gives update equations below. For $\mathrm{Y} \in \{\mathrm{C}, \mathrm{J}_1, \mathrm{I}_1\}$,

$$\ddot{\mu}_{\mathrm{Y}} = \frac{\sum_{s_1\in\mathbb{S}_1} \bar{\omega}_{\mathrm{Y}}(s_1)\bar{m}_{\mathrm{Y}}(s_1, \bar{\Delta}_{\mathrm{Y}}) + \sum_{s_2\in\mathbb{S}_2} \bar{\nu}_{\mathrm{Y}}(s_2)\bar{m}_{\mathrm{Y}}(s_2, \bar{\Delta}_{\mathrm{Y}})}{\sum_{s_1\in\mathbb{S}_1} \bar{\omega}_{\mathrm{Y}}(s_1) + \sum_{s_2\in\mathbb{S}_2} \bar{\nu}_{\mathrm{Y}}(s_2)}$$

$$\ddot{\Delta}_{\mathrm{Y}} = \frac{\sum_{s_1\in\mathbb{S}_1} \bar{\omega}_{\mathrm{Y}}(s_1)\bar{d}_{\mathrm{Y}}(s_1, \ddot{\mu}_{\mathrm{Y}}) + \sum_{s_2\in\mathbb{S}_2} \bar{\nu}_{\mathrm{Y}}(s_2)\bar{d}_{\mathrm{Y}}(s_2, \ddot{\mu}_{\mathrm{Y}})}{\sum_{s_1\in\mathbb{S}_1} \bar{\omega}_{\mathrm{Y}}(s_1)\xi_2(s_1, \bar{\theta}_{\mathrm{Y}}) + \sum_{s_2\in\mathbb{S}_2} \bar{\nu}_{\mathrm{Y}}(s_2)\xi_2(s_2, \bar{\theta}_{\mathrm{Y}})}$$

$$\ddot{\Gamma}_{\mathrm{Y}} = \frac{\sum_{s_1\in\mathbb{S}_1} \bar{\omega}_{\mathrm{Y}}(s_1)\bar{g}_{\mathrm{Y}}\left(s_1, \ddot{\mu}_{\mathrm{Y}}, \ddot{\Delta}_{\mathrm{Y}}\right) + \sum_{s_2\in\mathbb{S}_2} \bar{\nu}_{\mathrm{Y}}(s_2)\bar{g}_{\mathrm{Y}}\left(s_2, \ddot{\mu}_{\mathrm{Y}}, \ddot{\Delta}_{\mathrm{Y}}\right)}{\sum_{s_1\in\mathbb{S}_1} \bar{\omega}_{\mathrm{Y}}(s_1) + \sum_{s_2\in\mathbb{S}_2} \bar{\nu}_{\mathrm{Y}}(s_2)},$$

Table 3. Useful quantities for the parameter update equations. The quantities accented with $^-$ have the current estimates of all parameters, contained in $\bar{\zeta}$, as an implicit argument. Component specific quantities are subscripted by the component placeholder X or Y. $\mathrm{X} \in \mathbf{X} = \{\mathrm{C}, \mathrm{J}_1, \mathrm{I}_1\}$ and $\mathrm{Y} \in \mathbf{Y} = \{\mathrm{C}, \mathrm{J}_1, \mathrm{J}_2, \mathrm{I}_1, \mathrm{I}_2\}$. Parameters δ, Δ and Γ are related to the canonical SN parameters σ and λ as per Table 1. $\mathrm{TN}_+(\alpha, \psi^2)$ represents truncated normal distribution truncated below 0; α and ψ^2 are the location and scale parameters, respectively. $\mathbb{E}$ represents the expectation operator. $\frac{\phi}{\Phi}(\alpha/\psi)$ is the ratio of the pdf and the cdf of $\mathrm{N}(0,1)$ evaluated at α/ψ.

$$\bar{\omega}_{\mathrm{X}}(s_1) = \frac{\bar{w}_{\mathrm{X}} f_{\mathrm{SN}}(s_1; \bar{\theta}_{\mathrm{X}})}{\sum_{\mathrm{X}\in\mathbf{X}} \bar{w}_{\mathrm{X}} f_{\mathrm{SN}}(s_1; \bar{\theta}_{\mathrm{X}})}$$

$$\bar{\nu}_{\mathrm{Y}}(s_2) = \frac{\bar{v}_{\mathrm{Y}} f_{\mathrm{SN}}(s_2; \bar{\theta}_{\mathrm{Y}})}{\sum_{\mathrm{Y}\in\mathbf{Y}} \bar{v}_{\mathrm{Y}} f_{\mathrm{SN}}(s_2; \bar{\theta}_{\mathrm{Y}})}$$

$$\bar{m}_{\mathrm{Y}}(s, \Delta) = s - \xi_1(s, \bar{\theta}_{\mathrm{Y}})\Delta$$

$$\bar{d}_{\mathrm{Y}}(s, \mu) = \xi_1(s, \bar{\theta}_{\mathrm{Y}})(s - \mu)$$

$$\bar{g}_{\mathrm{Y}}(s, \mu, \Delta) = (s-\mu)^2 - 2\Delta\xi_1(s, \bar{\theta}_{\mathrm{Y}})(s-\mu) + \Delta^2\xi_2(s, \bar{\theta}_{\mathrm{Y}})$$

$$\text{For } T_s \sim \mathrm{TN}_+\left(\alpha = \frac{\delta}{\sigma}(s-\mu), \psi^2 = 1 - \delta^2\right),$$

$$\xi_1(s,\theta) = \mathbb{E}[T_s] = \alpha + \psi\frac{\phi}{\Phi}(\alpha/\psi)$$

$$\xi_2(s,\theta) = \mathbb{E}[T_s^2] = \alpha^2 + \psi^2 + \alpha\psi\frac{\phi}{\Phi}(\alpha/\psi)$$

where $\bar{m}_{\mathrm{Y}}(s,\Delta), \bar{d}_{\mathrm{Y}}(s,\mu)$ and $\bar{g}_{\mathrm{Y}}(s,\mu,\Delta)$ are defined in Table 3. For $\mathrm{Y} \in \{\mathrm{J}_2, \mathrm{I}_2\}$,

$$\ddot{\mu}_{\mathrm{Y}} = \frac{\sum_{s_2\in\mathbb{S}_2} \bar{\nu}_{\mathrm{Y}}(s_2)\bar{m}_{\mathrm{Y}}(s_2, \bar{\Delta}_{\mathrm{Y}})}{\sum_{s_2\in\mathbb{S}_2} \bar{\nu}_{\mathrm{Y}}(s_2)}$$

$$\ddot{\Delta}_{\mathrm{Y}} = \frac{\sum_{s_2\in\mathbb{S}_2} \bar{\nu}_{\mathrm{Y}}(s_2)\bar{d}_{\mathrm{Y}}(s_2, \ddot{\mu}_{\mathrm{Y}})}{\sum_{s_2\in\mathbb{S}_2} \bar{\nu}_{\mathrm{Y}}(s_2)\xi_2(s_2, \bar{\theta}_{\mathrm{Y}})}$$

$$\ddot{\Gamma}_{\mathrm{Y}} = \frac{\sum_{s_2\in\mathbb{S}_2} \bar{\nu}_{\mathrm{Y}}(s_2)\bar{g}_{\mathrm{Y}}\left(s_2, \ddot{\mu}_{\mathrm{Y}}, \ddot{\Delta}_{\mathrm{Y}}\right)}{\sum_{s_2\in\mathbb{S}_2} \bar{\nu}_{\mathrm{Y}}(s_2)}.$$

The new component parameters are guaranteed to not decrease the $\mathcal{Q}$-function; see Supplementary Materials. Note that for each component Y, its three parameters should be updated in the order, $\ddot{\mu}_{\mathrm{Y}} \to \ddot{\Delta}_{\mathrm{Y}} \to \ddot{\Gamma}_{\mathrm{Y}}$, due to dependencies among the equations.

4.4.3. Pairwise density constraints

To enforce the pairwise density constraints we developed a binary search procedure (Supplementary Materials) which is applied whenever a component density parameter ($\mu_{\mathrm{Y}}, \Delta_{\mathrm{Y}}$ or Γ_{Y}) is being updated with the new parameter from Section 4.4.2 as a candidate. Specifically, in case of μ_{Y}, if the new parameter, $\ddot{\mu}_{\mathrm{Y}}$, violates a pairwise density constraint involving component Y, a binary search is performed on the line segment connecting $\bar{\mu}_{\mathrm{Y}}$, and $\ddot{\mu}_{\mathrm{Y}}$ to find a feasible point, $\hat{\mu}_{\mathrm{Y}}$ (not violating the constraints), closest to $\ddot{\mu}_{\mathrm{Y}}$. A binary search is similarly performed when updating Δ_{Y} and Γ_{Y}. This approach is guaranteed to give feasible parameters at each iteration provided the first set of component parameters are feasible; see Supplementary Materials and Section 4.4.5. The parameters obtained from the binary search are also guaranteed to not decrease the $\mathcal{Q}$-function; see Supplementary Materials. Pairwise density constraints are efficiently evaluated as described in Supplementary Materials. Note that if $\ddot{\mu}_{\mathrm{Y}}$ ($\ddot{\Delta}_{\mathrm{Y}}$) is not feasible, then the feasible $\hat{\mu}_{\mathrm{Y}}$ ($\hat{\Delta}_{\mathrm{Y}}$), from the binary search, is used in the subsequent parameter updates of Δ_{Y} and Γ_{Y} in Section 4.4.2.

4.4.4. Weight updates under constraints

We update the weight parameters, $\{w_X\}_{X\in\mathbf{X}}$ and $\{v_Y\}_{Y\in\mathbf{Y}}$, by optimizing $\mathcal{Q}(\zeta|\bar{\zeta})$ under the weight constraint set B and the standard mixture constraints, $\sum_{X\in\mathbf{X}} w_X = 1$ and $\sum_{Y\in\mathbf{Y}} v_Y = 1$. Using the Karush–Kuhn–Tucker (KKT) approach for constrained optimization leads to the following Lagrangian objective.

$$\begin{aligned}\mathcal{O}(\boldsymbol{\zeta},\boldsymbol{\gamma},\boldsymbol{\eta}) = \mathcal{Q}(\zeta|\bar{\zeta}) &+ \gamma_1\Big(\sum\nolimits_{X\in\mathbf{X}} w_X - 1\Big) + \gamma_2\Big(\sum\nolimits_{Y\in\mathbf{Y}} v_Y - 1\Big)\\ &+ \eta_1(w_{J_1} + w_{I_1} - v'_C) + \eta_2(w_C + w_{I_1} - v'_{J_1})\\ &+ \eta_3(w_C + w_{J_1} - v'_{I_1}) + \eta_4(w_{J_1} - v'_{J_2})\\ &+ \eta_5(w_{I_1} - v'_{I_2}) + \eta_6(v'_{J_1} + v'_{I_1} + v_\Phi - w_C)\\ &+ \eta_7(v'_C + v'_{J_2} + v'_{I_1} + v_\Phi - w_{J_1})\\ &+ \eta_8(v'_C + v'_{J_1} + v'_{I_2} + v_\Phi - w_{I_1}), \end{aligned} \tag{3}$$

where $v'_Y = (1 - v_\Phi)v_Y$; $\boldsymbol{\gamma} = \{\gamma_1, \gamma_2\}$ and $\boldsymbol{\eta} = \{\eta_i\}_{i=1}^{8}$ are the KKT multipliers for the equality and inequality constraints, respectively. The KKT conditions lead to the following equations.

$$\begin{aligned}
\sum_{s_1\in\mathbb{S}_1} \frac{\bar{\omega}_C(s_1)}{w_C} + \gamma_1 + \eta_2 + \eta_3 - \eta_6 &= 0\\
\sum_{s_1\in\mathbb{S}_1} \frac{\bar{\omega}_{J_1}(s_1)}{w_{J_1}} + \gamma_1 + \eta_1 + \eta_3 + \eta_4 - \eta_7 &= 0\\
\sum_{s_1\in\mathbb{S}_1} \frac{\bar{\omega}_{I_1}(s_1)}{w_{I_1}} + \gamma_1 + \eta_1 + \eta_2 + \eta_5 - \eta_8 &= 0\\
\sum_{s_2\in\mathbb{S}_2} \frac{\bar{\nu}_C(s_2)}{v_C} + \gamma_2 - \eta_1 + \eta_7 + \eta_8 &= 0\\
\sum_{s_2\in\mathbb{S}_2} \frac{\bar{\nu}_{J_1}(s_2)}{v_{J_1}} + \gamma_2 - \eta_2 + \eta_6 + \eta_8 &= 0\\
\sum_{s_2\in\mathbb{S}_2} \frac{\bar{\nu}_{I_1}(s_2)}{v_{I_1}} + \gamma_2 - \eta_3 + \eta_6 + \eta_7 &= 0\\
\sum_{s_2\in\mathbb{S}_2} \frac{\bar{\nu}_{J_2}(s_2)}{v_{J_2}} + \gamma_2 - \eta_4 + \eta_7 &= 0\\
\sum_{s_2\in\mathbb{S}_2} \frac{\bar{\nu}_{I_2}(s_2)}{v_{I_2}} + \gamma_2 - \eta_5 + \eta_8 &= 0\\
w_C + w_{J_1} + w_{I_1} &= 1\\
v_C + v_{J_1} + v_{J_2} + v_{I_1} + v_{I_2} &= 1\\
\eta_1(w_{J_1} + w_{I_2} - v'_C) &= 0\\
\eta_2(w_C + w_{I_2} - v'_{J_1}) &= 0\\
\eta_3(w_C + w_{J_1} - v'_{I_2}) &= 0\\
\eta_4(w_{J_1} - v'_{J_2}) &= 0\\
\eta_5(w_{I_2} - v'_{I_2}) &= 0\\
\eta_6(v'_{J_1} + v'_{I_1} + v_\Phi - w_C) &= 0\\
\eta_7(v'_C + v'_{J_2} + v_{I_1} + v_\Phi - w_{J_1}) &= 0\\
\eta_8(v'_C + v'_{J_1} + v_{I_2} + v_\Phi - w_{I_1}) &= 0.
\end{aligned}$$

As per the KKT theory, the solution, $[\ddot{\boldsymbol{w}}, \ddot{\boldsymbol{v}}, \ddot{\boldsymbol{\gamma}}, \ddot{\boldsymbol{\eta}}]$, to the above system of equations gives the optimum mixing proportions, $[\ddot{\boldsymbol{w}}, \ddot{\boldsymbol{v}}]$, maximizing $\mathcal{Q}(\zeta|\bar{\zeta})$ and also satisfying the constraint set B and the standard mixing proportion constraints. Note that the last 8 equations, arising from the inequality constraints, require special consideration. For example, $\eta_1(w_{J_1} + w_{I_2} - v'_C) = 0$ implies that one of the two equations, $\eta_1 = 0$ and $w_{J_1} + w_{I_2} - v'_C = 0$, are satisfied. $\eta_1 = 0$ inactivates $v'_C \leq w_{J_1} + w_{I_2}$. It covers the case when the inequality $v'_C \leq w_{J_1} + w_{I_2}$ does not need to be enforced explicitly. The optimal feasible solution lies in the interior region of the inequality and already satisfies it. $\eta_1 \neq 0$ activates $v'_C = w_{J_1} + w_{I_2}$. It covers the case when the optimal feasible solution lies on the boundary of the inequality and consequently, it is enforced as an equality constraint. A practical implementation would require first solving the equations with $\eta_1 = 0$. If a feasible solution is obtained, it is optimal. If it violates the inequality, then the correct solution should lie on the boundary and consequently, $w_{J_1} + w_{I_2} - v'_C = 0$ is included in the system of equations to be solved.

Since there are multiple inequality constraints, finding the optimal solution would require an exhaustive search by solving all possible systems of equations obtained by equating each subset of $\{\eta_i\}_{i=1}^{8}$ to 0. This would lead to 256 different systems of equations. This approach is prohibitively expensive since the equations are solved in each iteration of the EM algorithm. As a practical solution, we adopt a greedy approach, where we first solve the system of equations without explicitly enforcing any inequality constraint; i.e., $\eta_i = 0$, $i = 1, 2 \ldots 8$. If none of the inequality constraints are violated, it gives the optimal solution. If any inequality constraint is violated, we run the system of equations with each of the violated constraints as active, separately; i.e., a single η parameter is non-zero. If a feasible solution is found, it gives the optimal solution. If no feasible solution is found, we run the system of equations again with two violated inequality constraints as active; i.e., exactly two η parameters are non-zero. Proceeding in this manner, we next check $3, 4, \ldots, 8$ active inequality constraints, if necessary. In all our experiments a feasible solution was obtained with a maximum of two active inequality constraints. Note that due to the inequality constraints, the updated weight parameters are not guaranteed to maintain the monotonicity of the $\mathcal{Q}$-function and the log-likelihood in each iteration. However, experimentally we still observe the log-likelihood to increase over multiple iterations.

4.4.5. Parameter initialization

To generate a diverse set of initial parameters, we adopted a random initialization approach. First a normal distribution is fitted to the top scores, with μ and σ being the fitted parameters. Then 5 points are sampled randomly from $\mathrm{N}(\mu, \sigma)$ and sorted. They are used to initialize the location parameters μ_C, μ_{J_1}, μ_{J_2}, μ_{I_1} and μ_{I_2} of the 5 SN components, assigned in that order. This approach makes it likely that the modes of the component densities follow the ordering described in Section 4.3.2. The scale parameter of the $Y \in \mathbf{Y}$ component, σ_Y, is uniformly picked from $[\sigma/4, \sigma]$. To initialize the skewness parameters, first a $\lambda_0 \in \{1, 2, 5\}$ is picked. Then the absolute value of component Y skewness parameter, λ_Y, is uniformly picked from $[1/\lambda_0, \lambda_0]$. The sign of the λ_C is initialized to be positive. The sign of $\lambda_{J_2}, \lambda_{I_1}$ and λ_{I_2} is initialized to be negative. λ_{J_1} is assigned a positive value in one initialization and a negative value in another. In this manner, two initializations with identical parameters, except the sign of λ_{J_1}, are obtained. If the initial parameters thus obtained violate the density constraints, they are discarded and resampled. Varying the value of λ_0 in $\{1, 2, 5\}$, 6 initializations are obtained. In each initialization, the mixing proportions w_C, w_{J_1} and w_{I_1} are set equally to 1/3. v_C is set to 0.001, since the second score is expected to have a small number of correct hits. $v_{J_1}, v_{J_2}, v_{I_1}$ and v_{I_2} are set equally to 0.999/4. Unlike the density constraints, it is not necessary for the initial parameters to satisfy the weight constraints.

We ran the above sampling procedure 40 times, resulting in $240 = 40 \times 6$ initilaizations. After running our algorithm once for

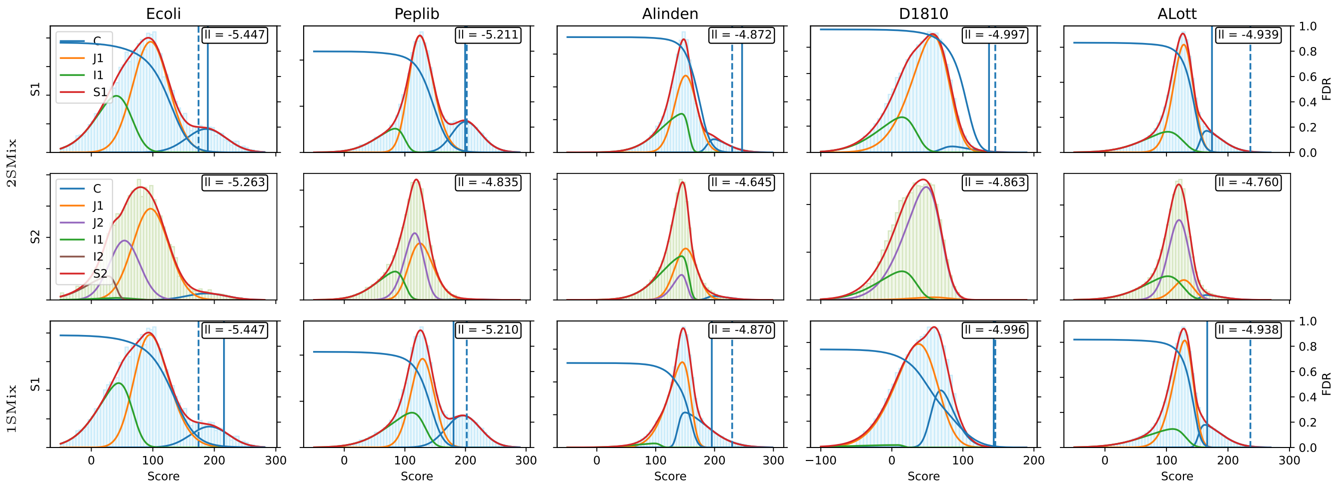

Fig. 1: The quality of the fit is visualized for five datasets (columns), with the remaining ones available in the Supplement. The data is shown as histograms, with blue representing top-ranked and green representing second-ranked PSM scores. The mixture components are plotted separately and each density is weighted by its mixture weight estimated by the model as described in Section 4. 1SMix and 2SMix are the one-sample and two-sample mixture models, respectively. The vertical solid line is showing the 1% FDR threshold given by the mixture model, while the vertical dashed line is showing the 1% FDR threshold given by TDA. The average log-likelihood of the fitted model on each sample is shown on the upper right corner. FDR curves are shown in blue with the y-axis and its scale shown on the right.

each initializations, we pick the solution attaining the maximum log-likelihood (Eq. 2) among them.

4.4.6. Single-sample model

We also considered a single-sample model that only incorporates the top score as a three component mixture; i.e., $S_1 \sim w_C\mathrm{SN}(\theta_C) + w_{J_1}\mathrm{SN}(\theta_{J_1}) + w_{I_1}\mathrm{SN}(\theta_{I_1})$. The weight constraints are not applicable to this model. However, the density constraints $f_C \succ f_{J_1}$ and $f_{J_1} \succ f_{I_1}$ are enforced. The parameter update equations for this model are given in Supplementary Materials. Similar parameter initialization approach and the same number of restarts as the two-sample model were used for a fair comparison.

4.4.7. FDR Estimation

The FDR of the fitted mixture model, at a given threshold τ, can be estimated as

$$\mathrm{FDR}(\tau) = \frac{w_{I_1} p(I_1 > \tau) + w_{J_1} p(J_1 > \tau)}{p(S_1 > \tau)}$$
$$\overset{est}{=} \frac{w_{I_1} S_{\mathrm{SN}}(\tau;\theta_{I_1}) + w_{J_1} S_{\mathrm{SN}}(\tau;\theta_{J_1})}{w_C S_{\mathrm{SN}}(\tau;\theta_C) + w_{I_1} S_{\mathrm{SN}}(\tau;\theta_{I_1}) + w_{J_1} S_{\mathrm{SN}}(\tau;\theta_{J_1})},$$

where $S_{\mathrm{SN}}(\tau;\theta) = 1 - F_{\mathrm{SN}}(\tau;\theta)$ is the SN distribution survival function; $F_{\mathrm{SN}}(\tau;\theta) = \Phi\left(\frac{\tau-\mu}{\sigma}\right) - 2O\left(\frac{\tau-\mu}{\sigma}, \lambda\right)$ is the SN distribution cdf; Φ is the cdf of $\mathrm{N}(0,1)$ and O is Owen's T function, computed approximately (Young and Minder, 1974).

5. Results

We carried out experiments on ten datasets (Table 2) and investigated the quality of fit, variance of estimation, and the number of identified peptides as a function of estimated FDR.

5.1. Quality of Modeling

The quality of fit is shown in Figure 1 for five selected datasets; for all datasets, please refer to the Supplementary Materials. In each case, the first two rows show the results of the two-sample model; i.e., joint modeling of the distributions of the top-ranked PSMs (blue histogram) and the second-ranked PSMs (green histogram). The third row shows the one-sample approach; i.e., when only the distribution of top-ranked PSMs is considered. Overall, the fit is excellent, indicating that the skew normal distribution was a reasonable choice for modeling competition between PSMs, which is a theoretically grounded result (Arellano-Valle *et al.*, 2006).

Compared to the TDA, the two-sample model gives competitive results. The 1% FDR threshold, shown by vertical lines in Figure 1, is similar in four out of ten datasets (D1810, Peplib, RPA, QE). For other datasets, the two-sample method gives different 1% FDR threshold, which is sometimes more permissive and other times more strict than the TDA threshold. By visual inspection and observation of some MS/MS spectra, we concluded that the two-sample solution may in fact be advantageous. The ALott dataset is an interesting case as our model leads to a more permissive FDR estimation. We have inspected multiple PSM identifications and concluded that the FDR = 1% threshold may in fact be closer to the one estimated by our method because, at least in some cases, the experimental spectra appear to be mixtures of two different pairs of cross-linked peptides with very similar total masses. Other cases of high-scoring second PSMs appear to correspond to the top PSMs with even higher scores. This dependency in the latter case cannot be easily modeled by the mixtures of distributions and is a limitation of our approach.

The one-sample model is providing relatively good results, often with an even better log-likelihood than the two-sample model; however, the lack of the second sample in parameter learning leads to a considerable error in distribution placement and, consequently, FDR estimation. One such example is the Alinden data where the 1% FDR threshold of 195 is lower than the TDA's threshold of 230 and the two-sample model's threshold of 247. This result is problematic because the tail area of the score distribution of the second-ranked PSMs (that this model is not considering) above 195 is quite large and cannot be attributed to the correct PSMs. Therefore, this model is clearly inferior to the two-sample model in its quality of fit.

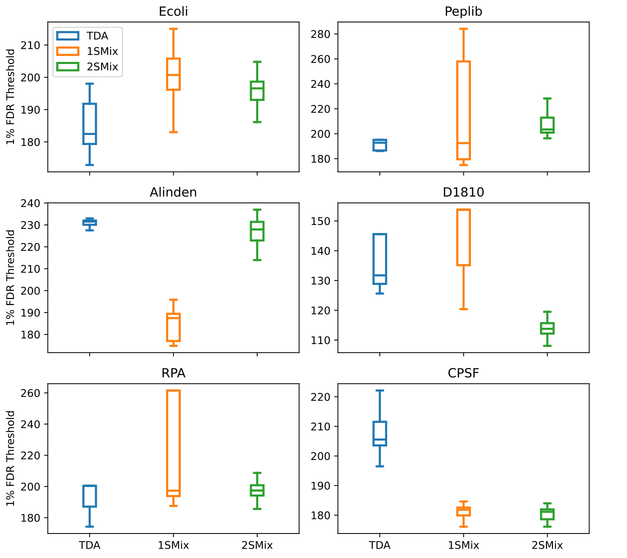

Fig. 2: Stability of the 1% FDR threshold estimation on selected datasets. The stability of the methods was compared using 50 bootstrap samples on which the 1% FDR thresholds were estimated, as shown on the y-axis of each plot. The larger spread of a boxplot indicates lower stability. Bootstrapping was performed on the set of original mass spectra.

We used constraints to control the relative placement of the component distributions. For example, the correct component should take the rightmost part of the score distribution of the top-ranked PSMs as they should have higher mean values than incorrect or partially incorrect matches. Similarly, when both the top hits and second hits are partially incorrect, they should have similar shape of the distribution. While this was not enforced by the constraints, we observed that the first and second partially incorrect distributions indeed have similar shapes as well as that the second partially incorrect distribution had a lower mean than the first partially incorrect distribution.

5.2. Variance of the FDR Threshold

We used fifty bootstrapping (Efron and Tibshirani, 1986) experiments to study the variance of estimated 1% FDR thresholds (Figure 2). In most cases the two-sample model gives a stable threshold, although TDA performs well on large datasets such as Alinden. This is expected and is the case when TDA assumptions are likely to be satisfied (Elias and Gygi, 2007). The larger variance of the 1% FDR thresholds in the one-sample mixture suggests an identifiability issue, which is mitigated by incorporating the second sample and the weight constraints.

5.3. Spectral Identifications

Figure 3 shows the number of identified PSMs as a function of estimated FDR thresholds. We first observe that our model generates smooth curves, which is desirable. In some cases, the two-sample model shows great agreement with the TDA suggesting that decoy data may not give any information that is not already incorporated by the DFA. Examples of such cases are Ecoli and D1810 datasets. Interestingly, however, the bootstrapping results on these datasets show increased stability of DFA and suggest that the DFA should be the preferred choice on such data.

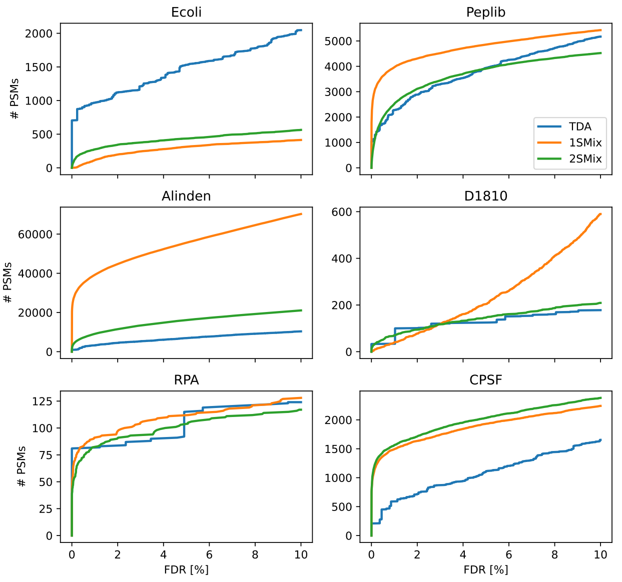

Fig. 3: Identified PSMs on selected datasets at specific FDR levels.

6. Discussion

Accurate false discovery rate estimation is a key to biological discovery (Nesvizhskii, 2010; Aggarwal and Yadav, 2016) and is integral to protein identification methodology (Li and Radivojac, 2012; Serang and Noble, 2012) and protein function studies (Sinz, 2003, 2006). However, the field is confronted with computational and statistical challenges and the quality of methods are difficult to evaluate owing to the lack of the ground truth associated with experimental spectra.

To the best of our knowledge, this work is the first to propose a decoy-free FDR estimation in XL-MS/MS. We have accomplished this by modeling top-ranked and second-ranked PSM score distributions as multi-component mixtures of (latent) skew normal distributions with shared parameters. We formulated the problem as a constrained maximum likelihood optimization and then derived an EM algorithm to learn model parameters from data. We extensively evaluated this method to show that the low-variance quality FDR estimation can be achieved without decoy data. The proposed algorithm is a nontrivial generalization of the multi-sample decoy-free approaches we developed for traditional MS/MS (Peng *et al.*, 2020) although the use of multiple components to model incorrect PSM scores in XL-MS/MS required constrained optimization and a far more complex solution. However, as before, modeling of the score distribution of the second-ranked PSMs has stabilized learning, and helped avoid target-decoy competition, leading to an accurate inference procedure with significant run-time savings. The reasoning behind this algorithm can be further applied to other large search-space MS/MS scenarios, including de novo searches (Dancik *et al.*, 1999), searches of semi-tryptic (Alves *et al.*, 2008) and post-translationally modified (Fu, 2012) peptides, as well as to metaproteomics searches (Heyer *et al.*, 2017).

7. Acknowledgments

We gratefully acknowledge the assistance and expert advice provided by the OpenPepXL team.

References

Aggarwal, S. and Yadav, A. K. (2016). False discovery rate estimation in proteomics. *Methods Mol Biol*, **1362**, 119–128.

Alves, P. *et al.* (2008). Fast and accurate identification of semi-tryptic peptides in shotgun proteomics. *Bioinformatics*, **24**(1), 102–109.

Arellano-Valle, R. B. *et al.* (2006). A unified view on skewed distributions arising from selections. *Can J Stat*, **34**(4), 581–601.

Azzalini, A. (1985). A class of distributions which includes the normal ones. *Scand J Stat*, **12**(2), 171–178.

Budnik, B. *et al.* (2018). SCoPE-MS: mass spectrometry of single mammalian cells quantifies proteome heterogeneity during cell differentiation. *Genome Biol*, **19**(1), 161.

Burger, T. (2018). Gentle introduction to the statistical foundations of false discovery rate in quantitative proteomics. *J Proteome Res*, **17**(1), 12–22.

Choi, H. and Nesvizhskii, A. I. (2008). False discovery rates and related statistical concepts in mass spectrometry-based proteomics. *J Proteome Res*, **7**(1), 47–50.

Cooper, B. (2011). The problem with peptide presumption and low Mascot scoring. *J Proteome Res*, **10**(3), 1432–1435.

Cooper, B. (2012). The problem with peptide presumption and the downfall of target-decoy false discovery rates. *Anal Chem*, **84**(22), 9663–9667.

Dancik, V. *et al.* (1999). De novo peptide sequencing via tandem mass spectrometry. *J Comput Biol*, **6**(3-4), 327–342.

Danilova, Y. *et al.* (2019). Bias in false discovery rate estimation in mass-spectrometry-based peptide identification. *J Proteome Res*, **18**(5), 2354–2358.

Efron, B. and Tibshirani, R. (1986). Bootstrap methods for standard errors, confidence intervals, and other measures of statistical accuracy. *Stat Sci*, **1**(1), 54–75.

Elias, J. E. and Gygi, S. P. (2007). Target-decoy search strategy for increased confidence in large-scale protein identifications by mass spectrometry. *Nat Methods*, **4**(3), 207–214.

Frank, A. and Pevzner, P. (2005). PepNovo: de novo peptide sequencing via probabilistic network modeling. *Anal Chem*, **77**(4), 964–973.

Fu, Y. (2012). Bayesian false discovery rates for post-translational modification proteomics. *Stat Interface*, **5**(1), 47–59.

Gupta, N. *et al.* (2011). Target-decoy approach and false discovery rate: when things may go wrong. *J Am Soc Mass Spectrom*, **22**(7), 1111–1120.

He, K. *et al.* (2015). A theoretical foundation of the target-decoy search strategy for false discovery rate control in proteomics. *arXiv preprint arXiv:1501.00537*.

Henze, N. (1986). A probabilistic representation of the 'skew-normal' distribution. *Scand J Stat*, **13**(4), 271–275.

Heyer, R. *et al.* (2017). Challenges and perspectives of metaproteomic data analysis. *J Biotechnol*, **261**, 24–36.

Hoopmann, M. R. *et al.* (2015). Kojak: efficient analysis of chemically cross-linked protein complexes. *J Proteome Res*, **14**(5), 2190–2198.

Jeong, K. *et al.* (2012). False discovery rates in spectral identification. *BMC Bioinformatics*, **13**(Suppl 16), S2.

Ji, C. *et al.* (2016). XLSearch: a probabilistic database search algorithm for identifying cross-linked peptides. *J Proteome Res*, **15**(6), 1830–1841.

Kall, L. *et al.* (2008a). Assigning significance to peptides identified by tandem mass spectrometry using decoy databases. *J Proteome Res*, **7**(1), 29–34.

Kall, L. *et al.* (2008b). Posterior error probabilities and false discovery rates: two sides of the same coin. *J Proteome Res*, **7**(1), 40–44.

Keller, A. *et al.* (2002). Empirical statistical model to estimate the accuracy of peptide identifications made by MS/MS and database search. *Anal Chem*, **74**(20), 5383–5392.

Kim, S. and Pevzner, P. A. (2014). MS-GF+ makes progress towards a universal database search tool for proteomics. *Nat Commun*, **5**, 5277.

Kong, A. T. *et al.* (2017). MSFragger: ultrafast and comprehensive peptide identification in mass spectrometry-based proteomics. *Nat Methods*, **14**(5), 513–520.

Li, Q. (2008). *Statistical methods for peptide and protein identification using mass spectrometry*. Ph.D. thesis.

Li, S. *et al.* (2015). An integrated platform for isolation, processing, and mass spectrometry-based proteomic profiling of rare cells in whole blood. *Mol Cell Proteomics*, **14**(6), 1672–1683.

Li, Y. F. and Radivojac, P. (2012). Computational approaches to protein inference in shotgun proteomics. *BMC Bioinformatics*, **13**(Suppl 16), S4.

Lin, T. I. (2009). Maximum likelihood estimation for multivariate skew normal mixture models. *J Multivar Anal*, **100**(2), 257–265.

Lin, T. I. *et al.* (2007). Finite mixture modelling using the skew normal distribution. *Stat Sinica*, **17**(3), 909.

Meng, X.-L. and Rubin, D. B. (1993). Maximum likelihood estimation via the ECM algorithm: a general framework. *Biometrika*, **80**(2), 267–278.

Nesvizhskii, A. I. (2010). A survey of computational methods and error rate estimation procedures for peptide and protein identification in shotgun proteomics. *J Proteomics*, **73**(11), 2092–2123.

Netz, E. *et al.* (2020). OpenPepXL: an open-source tool for sensitive identification of cross-linked peptides in XL-MS. *Mol Cell Proteomics*, **19**(12), 2157–2168.

Peng, Y. *et al.* (2020). New mixture models for decoy-free false discovery rate estimation in mass spectrometry proteomics. *Bioinformatics*, **36**(Suppl_2), i745–i753.

Perez-Riverol, Y. *et al.* (2022). The PRIDE database resources in 2022: a hub for mass spectrometry-based proteomics evidences. *Nucleic Acids Res*, **50**(D1), D543–D552.

Perkins, D. N. *et al.* (1999). Probability-based protein identification by searching sequence databases using mass spectrometry data. *Electrophoresis*, **20**(18), 3551–3567.

Piersimoni, L. *et al.* (2021). Cross-linking mass spectrometry for investigating protein conformations and protein-protein interactions – a method for all seasons. *Chem Rev*, **122**(8), 7500–7531.

Rappsilber, J. (2011). The beginning of a beautiful friendship: cross-linking/mass spectrometry and modelling of proteins and multi-protein complexes. *J Struct Biol*, **173**(3), 530–540.

Rinner, O. *et al.* (2008). Identification of cross-linked peptides from large sequence databases. *Nat Methods*, **5**(4), 315–318.

Serang, O. and Noble, W. S. (2012). A review of statistical methods for protein identification using tandem mass spectrometry. *Stat Interface*, **5**(1), 3–20.

Sinz, A. (2003). Chemical cross-linking and mass spectrometry for mapping three-dimensional structures of proteins and protein complexes. *J Mass Spectrom*, **38**(12), 1225–1237.

Sinz, A. (2006). Chemical cross-linking and mass spectrometry to map three-dimensional protein structures and protein-protein interactions. *Mass Spectrom Rev*, **25**(4), 663–682.

Steen, H. and Mann, M. (2004). The ABC's (and XYZ's) of peptide sequencing. *Nat Rev Mol Cell Biol*, **5**(9), 699–711.

Storey, J. D. (2002). A direct approach to false discovery rate. *J R Statist Soc B*, **64**(3), 479–498.

Walzthoeni, T. *et al.* (2012). False discovery rate estimation for cross-linked peptides identified by mass spectrometry. *Nat Methods*, **9**(9), 901–903.

Yang, B. *et al.* (2012). Identification of cross-linked peptides from complex samples. *Nat Methods*, **9**(9), 904–906.

Yates, J. R., r. *et al.* (1995). Method to correlate tandem mass spectra of modified peptides to amino acid sequences in the protein database. *Anal Chem*, **67**(8), 1426–1436.

Young, J. C. and Minder, C. E. (1974). Algorithm AS 76: an integral useful in calculating non-central t and bivariate normal probabilities. *J R Statist Soc C*, **23**(3), 455–457.

Yu, C. and Huang, L. (2018). Cross-linking mass spectrometry (XL-MS): an emerging technology for interactomics and structural biology. *Anal Chem*, **90**(1), 144–165.
