# Supplementary material for: An algorithm for decoy-free false discovery rate estimation in XL-MS/MS proteomics
Source: Bioinformatics. 2024 Jun 28;40(Suppl 1):i428–36. doi: 10.1093/bioinformatics/btae233 (PMC11256928; doi:10.1093/bioinformatics/btae233)
Supplement: btae233_Supplementary_Data [file btae233_supplementary_data.zip › btae233_Supplementary_Data/Radivojac.246.sup.1.pdf]

Supplementary Materials

March 20, 2024

A Derivation of constraint set B

Let $r_{\mathrm{C}}, r_{\mathrm{J}_1}$ and r_{I_1} be the proportaion of the top scores with missing second score that come from C, J_1 and I_1, respectively, where, for $\mathrm{X} \in \mathbf{X} = \{\mathrm{C}, \mathrm{J}_1, \mathrm{I}_1\}$, $r_{\mathrm{X}} > 0$ and $\sum_{\mathrm{X}} r_{\mathrm{X}} = 1$. The proportion of top scores coming from X and having a second score is $w_{\mathrm{X}} - r_{\mathrm{X}} v_{\Phi}$. The proportion of observed second scores coming from Y ($\mathrm{Y} \in \mathbf{Y} = \{\mathrm{C}, \mathrm{J}_1, \mathrm{I}_1, \mathrm{J}_2, \mathrm{J}_1\}$) among all observed and missing second scores is $(1 - v_{\Phi}) v_{\mathrm{Y}}$. Uptading the contraint set A by replacing all occurances of w_{X} and v_{Y} by $w_{\mathrm{X}} - r_{\mathrm{X}} v_{\Phi}$ and $(1 - v_{\Phi}) v_{\mathrm{Y}}$ we get an updated constraint set based only on the spectrum where both the scores are available. For example, $w_{\mathrm{C}} \leq v_{\mathrm{J}_1} + v_{\mathrm{I}_1}$ is replaced by $w_{\mathrm{C}} - r_{\mathrm{C}} v_{\Phi} \leq (1 - v_{\Phi})(v_{\mathrm{J}_1} + v_{\mathrm{I}_1})$ and $v_{\mathrm{C}} \leq w_{\mathrm{J}_1} + w_{\mathrm{I}_1}$ is replaced by $(1 - v_{\Phi}) v_{\mathrm{C}} \leq w_{\mathrm{J}_1} + w_{\mathrm{I}_1} - (r_{\mathrm{J}_1} + r_{\mathrm{I}_1}) v_{\Phi}$. Since r_{X} is an unknown quantity, we further modify the new constraints by using $r_{\mathrm{X}} = 1$ ($r_{\mathrm{X}} = 0$) when it appears on the left (right) hand side, since it minimizes (maximizes) $w_{\mathrm{X}} - r_{\mathrm{X}} v_{\Phi}$, making the constraints looser and valid wihtout any assumptions on the ditribution of top scores with missing second score. The two contraints in the example above get transformed to $w_{\mathrm{C}} - v_{\Phi} \leq (1 - v_{\Phi})(v_{\mathrm{J}_1} + v_{\mathrm{I}_1})$ and $(1 - v_{\Phi}) v_{\mathrm{C}} \leq w_{\mathrm{J}_1} + w_{\mathrm{I}_1}$. All the constraints modified in this manner are given as constraint set B with $v'_{\mathrm{Y}} = (1 - v_{\Phi}) v_{\mathrm{Y}}$.

B Monotonicity of the ECM algorithm and the binary search

The ECM algorithm based component parameter updates are derived by optimizing the $\mathcal{Q}(\boldsymbol{\zeta}|\bar{\boldsymbol{\zeta}})$ one parameter at a time. When optimizing $\mathcal{Q}(\boldsymbol{\zeta}|\bar{\boldsymbol{\zeta}})$ w.r.t. component Y parameters, $\boldsymbol{\theta}_{\mathrm{Y}} = \{\mu_{\mathrm{Y}}, \Delta_{\mathrm{Y}}, \Gamma_{\mathrm{Y}}\}$, the other components' parameters and the weight parameters in $\boldsymbol{\zeta}$ can be ignored as they appear in additive terms, constant w.r.t. $\boldsymbol{\theta}_{\mathrm{Y}}$. However, note that all parameters in the current parameter set $\bar{\boldsymbol{\zeta}}$ still play a role in component Y parameter updates. In effect optimizing the $\mathcal{Q}$-function w.r.t. component Y parameters reduces to the optimization of $\mathcal{Q}(\mu_{\mathrm{Y}}, \Delta_{\mathrm{Y}}, \Gamma_{\mathrm{Y}}|\bar{\boldsymbol{\zeta}})$ as defined below. For $\mathrm{Y} \in \{\mathrm{C}, \mathrm{J}_1, \mathrm{I}_1\}$,

$$\begin{aligned}
\mathcal{Q}(\mu_{\mathrm{Y}}, \Delta_{\mathrm{Y}}, \Gamma_{\mathrm{Y}}|\bar{\boldsymbol{\zeta}}) = &-\frac{1}{2} \sum_{s_1 \in \mathbb{S}_1} \bar{\omega}_{\mathrm{X}}(s_1) \left(\log \Gamma_{\mathrm{Y}} + \frac{(s_1 - \mu_{\mathrm{Y}})^2 - 2\xi_1(s_1, \bar{\boldsymbol{\zeta}})(s_1 - \mu_{\mathrm{Y}})\Delta_{\mathrm{Y}} + \xi_2(s_1, \bar{\boldsymbol{\zeta}})(\Delta_{\mathrm{Y}}^2 + \Gamma_{\mathrm{Y}})}{\Gamma_{\mathrm{Y}}} \right) \\
&- \frac{1}{2} \sum_{s_2 \in \mathbb{S}_2} \bar{\nu}_{\mathrm{Y}}(s_2) \left(\log \Gamma_{\mathrm{Y}} + \frac{(s_2 - \mu_{\mathrm{Y}})^2 - 2\xi_1(s_2, \bar{\boldsymbol{\zeta}})(s_2 - \mu_{\mathrm{Y}})\Delta_{\mathrm{Y}} + \xi_2(s_2, \bar{\boldsymbol{\zeta}})(\Delta_{\mathrm{Y}}^2 + \Gamma_{\mathrm{Y}})}{\Gamma_{\mathrm{Y}}} \right).
\end{aligned}$$

And for $\mathrm{Y} \in \{\mathrm{J}_2, \mathrm{I}_2\}$,

$$\mathcal{Q}(\mu_{\mathrm{Y}}, \Delta_{\mathrm{Y}}, \Gamma_{\mathrm{Y}}|\bar{\boldsymbol{\zeta}}) = -\frac{1}{2} \sum_{s_2 \in \mathbb{S}_2} \bar{\nu}_{\mathrm{Y}}(s_2) \left(\log \Gamma_{\mathrm{Y}} + \frac{(s_2 - \mu_{\mathrm{Y}})^2 - 2\xi_1(s_2, \bar{\boldsymbol{\zeta}})(s_2 - \mu_{\mathrm{Y}})\Delta_{\mathrm{Y}} + \xi_2(s_2, \bar{\boldsymbol{\zeta}})(\Delta_{\mathrm{Y}}^2 + \Gamma_{\mathrm{Y}})}{\Gamma_{\mathrm{Y}}} \right).$$

The new parameters $\ddot{\mu}_{\mathrm{Y}}, \ddot{\Delta}_{\mathrm{Y}}$ and $\ddot{\Gamma}_{\mathrm{Y}}$ from the ECM parameter update equations are obtained as the unique stationary points (where the first derivative is 0) of $\mathcal{Q}(\mu_{\mathrm{Y}}, \bar{\Delta}_{\mathrm{Y}}, \bar{\Gamma}_{\mathrm{Y}}|\bar{\boldsymbol{\zeta}})$, $\mathcal{Q}(\ddot{\mu}_{\mathrm{Y}}, \Delta_{\mathrm{Y}}, \bar{\Gamma}_{\mathrm{Y}}|\bar{\boldsymbol{\zeta}})$ and $\mathcal{Q}(\ddot{\mu}_{\mathrm{Y}}, \ddot{\Delta}_{\mathrm{Y}}, \Gamma_{\mathrm{Y}}|\bar{\boldsymbol{\zeta}})$ as a single variable function of $\mu_{\mathrm{Y}}, \Delta_{\mathrm{Y}}$ and Γ_{Y}, respectively.

$\mathcal{Q}(\mu_{\mathrm{Y}}, \bar{\Delta}_{\mathrm{Y}}, \bar{\Gamma}_{\mathrm{Y}}|\bar{\boldsymbol{\zeta}})$ and $\mathcal{Q}(\ddot{\mu}_{\mathrm{Y}}, \Delta_{\mathrm{Y}}, \bar{\Gamma}_{\mathrm{Y}}|\bar{\boldsymbol{\zeta}})$ as functions μ_{Y} and Δ_{Y}, respectively, are concave, since their second derivatives are not positive everywhere. Consequently, $\ddot{\mu}_{\mathrm{Y}}$ and $\ddot{\Delta}_{\mathrm{Y}}$ are the global maximizers.

Now $\mathcal{Q}(\ddot{\mu}_\mathrm{Y}, \ddot{\Delta}_\mathrm{Y}, \Gamma_\mathrm{Y}|\bar{\boldsymbol{\zeta}})$ is not a concave function of Γ_Y, however, it can be expressed as a difference of two convex functions as follows.

$$\begin{aligned}
\mathcal{Q}(\ddot{\mu}_\mathrm{Y}, \ddot{\Delta}_\mathrm{Y}, \Gamma_\mathrm{Y}|\bar{\boldsymbol{\zeta}}) &= -\frac{1}{2}\sum_{s_1\in\mathbb{S}_1} \bar{\omega}_\mathrm{X}(s_1)\left(\log\Gamma_\mathrm{Y} + \frac{(s_1-\ddot{\mu}_\mathrm{Y})^2 - 2\xi_1(s_1,\bar{\boldsymbol{\zeta}})(s_1-\ddot{\mu}_\mathrm{Y})\ddot{\Delta}_\mathrm{Y} + \xi_2(s_1,\bar{\boldsymbol{\zeta}})(\ddot{\Delta}_\mathrm{Y}^2+\Gamma_\mathrm{Y})}{\Gamma_\mathrm{Y}}\right)\\
&\quad -\frac{1}{2}\sum_{s_2\in\mathbb{S}_2} \bar{\nu}_\mathrm{Y}(s_2)\left(\log\Gamma_\mathrm{Y} + \frac{(s_2-\ddot{\mu}_\mathrm{Y})^2 - 2\xi_1(s_2,\bar{\boldsymbol{\zeta}})(s_2-\ddot{\mu}_\mathrm{Y})\ddot{\Delta}_\mathrm{Y} + \xi_2(s_2,\bar{\boldsymbol{\zeta}})(\ddot{\Delta}_\mathrm{Y}^2+\Gamma_\mathrm{Y})}{\Gamma_\mathrm{Y}}\right)\\
&= \frac{1}{2}\left(\sum_{s_1\in\mathbb{S}_1}\bar{\omega}_\mathrm{X}(s_1) + \sum_{s_2\in\mathbb{S}_2}\bar{\nu}_\mathrm{Y}(s_2)\right)\left(\log {}^{1}\!/_{\Gamma_\mathrm{Y}} - {}^{\ddot{\Gamma}_\mathrm{Y}}\!/_{\Gamma_\mathrm{Y}}\right) + \text{constant}.
\end{aligned}$$

In the equation above $g(\Gamma_\mathrm{Y}) = {}^{\ddot{\Gamma}_\mathrm{Y}}\!/_{\Gamma_\mathrm{Y}}$ and $h(\Gamma_\mathrm{Y}) = \log {}^{1}\!/_{\Gamma_\mathrm{Y}}$ are convex functions, since their second derivatives are non-negative everywhere. Now based on Definition 2.2.2 in [1], the ϵ-subdifferential of g and h at $\ddot{\Gamma}_\mathrm{Y}$ is given by

$$\partial_\epsilon g(\ddot{\Gamma}_\mathrm{Y}) = \begin{cases} \left(-\infty, -\frac{1}{\Gamma_\mathrm{Y}} + \frac{\epsilon}{(\Gamma_\mathrm{Y}-\ddot{\Gamma}_\mathrm{Y})}\right] & \text{when } \Gamma_\mathrm{Y} \geq \ddot{\Gamma}_\mathrm{Y}\\ \left[-\frac{1}{\Gamma_\mathrm{Y}} + \frac{\epsilon}{(\Gamma_\mathrm{Y}-\ddot{\Gamma}_\mathrm{Y})}, \infty\right) & \text{when } \Gamma_\mathrm{Y} < \ddot{\Gamma}_\mathrm{Y}\end{cases}$$

$$\partial_\epsilon h(\ddot{\Gamma}_\mathrm{Y}) = \begin{cases} \left(-\infty, \frac{\log\left({}^{\ddot{\Gamma}_\mathrm{Y}}/_{\Gamma_\mathrm{Y}}\right)}{(\Gamma_\mathrm{Y}-\ddot{\Gamma}_\mathrm{Y})} + \frac{\epsilon}{(\Gamma_\mathrm{Y}-\ddot{\Gamma}_\mathrm{Y})}\right] & \text{when } \Gamma_\mathrm{Y} \geq \ddot{\Gamma}_\mathrm{Y}\\ \left[\frac{\log\left({}^{\ddot{\Gamma}_\mathrm{Y}}/_{\Gamma_\mathrm{Y}}\right)}{(\Gamma_\mathrm{Y}-\ddot{\Gamma}_\mathrm{Y})} + \frac{\epsilon}{(\Gamma_\mathrm{Y}-\ddot{\Gamma}_\mathrm{Y})}, \infty\right) & \text{when } \Gamma_\mathrm{Y} < \ddot{\Gamma}_\mathrm{Y}\end{cases}$$

Now since $\log\left({}^{\ddot{\Gamma}_\mathrm{Y}}/_{\Gamma_\mathrm{Y}}\right) \leq \frac{\ddot{\Gamma}_\mathrm{Y}}{\Gamma_\mathrm{Y}} - 1$,

$$\begin{aligned}
\frac{\log\left({}^{\ddot{\Gamma}_\mathrm{Y}}/_{\Gamma_\mathrm{Y}}\right)}{\Gamma_\mathrm{Y}-\ddot{\Gamma}_\mathrm{Y}} &\leq -\frac{1}{\Gamma_\mathrm{Y}} \quad \text{when } \Gamma_\mathrm{Y} \geq \ddot{\Gamma}_\mathrm{Y}\\
\frac{\log\left({}^{\ddot{\Gamma}_\mathrm{Y}}/_{\Gamma_\mathrm{Y}}\right)}{\Gamma_\mathrm{Y}-\ddot{\Gamma}_\mathrm{Y}} &\geq -\frac{1}{\Gamma_\mathrm{Y}} \quad \text{when } \Gamma_\mathrm{Y} < \ddot{\Gamma}_\mathrm{Y}.
\end{aligned}$$

Thus $\partial_\epsilon h(\ddot{\Gamma}_\mathrm{Y}) \subseteq \partial_\epsilon g(\ddot{\Gamma}_\mathrm{Y})$ and, consequently from Theorem 2.3.1 in [1], $g(\Gamma_\mathrm{Y}) - h(\Gamma_\mathrm{Y})$ has a global minimizer at $\ddot{\Gamma}_\mathrm{Y}$. It follows that $h(\Gamma_\mathrm{Y}) - g(\Gamma_\mathrm{Y})$ and conseqently $\mathcal{Q}(\ddot{\mu}_\mathrm{Y}, \ddot{\Delta}_\mathrm{Y}, \Gamma_\mathrm{Y}|\bar{\boldsymbol{\zeta}})$, has a global maximizer at $\ddot{\Gamma}_\mathrm{Y}$.

Since $\ddot{\mu}_\mathrm{Y}, \ddot{\Delta}_\mathrm{Y}$ and $\ddot{\Gamma}_\mathrm{Y}$ are global maximizers of $\mathcal{Q}(\mu_\mathrm{Y}, \bar{\Delta}_\mathrm{Y}, \bar{\Gamma}_\mathrm{Y}|\bar{\boldsymbol{\zeta}})$, $\mathcal{Q}(\ddot{\mu}_\mathrm{Y}, \Delta_\mathrm{Y}, \bar{\Gamma}_\mathrm{Y}|\bar{\boldsymbol{\zeta}})$ and $\mathcal{Q}(\ddot{\mu}_\mathrm{Y}, \ddot{\Delta}_\mathrm{Y}, \Gamma_\mathrm{Y}|\bar{\boldsymbol{\zeta}})$, respectively,

$$\begin{aligned}
\mathcal{Q}(\bar{\mu}_\mathrm{Y}, \bar{\Delta}_\mathrm{Y}, \bar{\Gamma}_\mathrm{Y}|\bar{\boldsymbol{\zeta}}) &\leq \mathcal{Q}(\ddot{\mu}_\mathrm{Y}, \bar{\Delta}_\mathrm{Y}, \bar{\Gamma}_\mathrm{Y}|\bar{\boldsymbol{\zeta}})\\
&\leq \mathcal{Q}(\ddot{\mu}_\mathrm{Y}, \ddot{\Delta}_\mathrm{Y}, \bar{\Gamma}_\mathrm{Y}|\bar{\boldsymbol{\zeta}})\\
&\leq \mathcal{Q}(\ddot{\mu}_\mathrm{Y}, \ddot{\Delta}_\mathrm{Y}, \ddot{\Gamma}_\mathrm{Y}|\bar{\boldsymbol{\zeta}}).
\end{aligned}$$

Thus the new component parameters from the ECM updates increase (precisely, do not decrease) the value of the $\mathcal{Q}$-function at each iteration.

The monotonicity of the $\mathcal{Q}$-function can be similarly established for the parameter updates from the binary search. $\mathcal{Q}(\mu_\mathrm{Y}, \bar{\Delta}_\mathrm{Y}, \bar{\Gamma}_\mathrm{Y}|\bar{\boldsymbol{\zeta}})$, $\mathcal{Q}(\ddot{\mu}_\mathrm{Y}, \Delta_\mathrm{Y}, \bar{\Gamma}_\mathrm{Y}|\bar{\boldsymbol{\zeta}})$ and $\mathcal{Q}(\ddot{\mu}_\mathrm{Y}, \ddot{\Delta}_\mathrm{Y}, \Gamma_\mathrm{Y}|\bar{\boldsymbol{\zeta}})$ each have a unique stationary point (where the first derivative is 0), $\ddot{\mu}_\mathrm{Y}, \ddot{\Delta}_\mathrm{Y}$ and $\ddot{\Gamma}_\mathrm{Y}$, respectively, which is the global maximizer. Furthermore, they do not have any singular points (where the first derivative is undefined), except for $\mathcal{Q}(\ddot{\mu}_\mathrm{Y}, \ddot{\Delta}_\mathrm{Y}, \Gamma_\mathrm{Y}|\bar{\boldsymbol{\zeta}})$ at $\Gamma_\mathrm{Y} = 0$, which is an endpoint. Thus none of them have a local minimum and an absolute minimum only exists at the endpoints. It follows that the three functions are unimodal with $\ddot{\mu}_\mathrm{Y}, \ddot{\Delta}_\mathrm{Y}$ and $\ddot{\Gamma}_\mathrm{Y}$ giving the modes. Thus any point on the line connecting $\bar{\mu}_\mathrm{Y}$ to $\ddot{\mu}_\mathrm{Y}$, $\bar{\Delta}_\mathrm{Y}$ to $\ddot{\Delta}_\mathrm{Y}$ and $\bar{\Gamma}_\mathrm{Y}$ to $\ddot{\Gamma}_\mathrm{Y}$ would not decrease the value of the corresponding $\mathcal{Q}$-function. Precisely, in case of μ_Y, binary search finds a feasible $\hat{\mu}_\mathrm{Y}$ on the line connecting $\bar{\mu}_\mathrm{Y}$ and $\ddot{\mu}_\mathrm{Y} = \operatorname{argmax}_{\mu_\mathrm{Y}} \mathcal{Q}(\mu_\mathrm{Y}, \bar{\Delta}_\mathrm{Y}, \bar{\Gamma}_\mathrm{Y}|\bar{\boldsymbol{\zeta}})$. Thus $\mathcal{Q}(\bar{\mu}_\mathrm{Y}, \bar{\Delta}_\mathrm{Y}, \bar{\Gamma}_\mathrm{Y}|\bar{\boldsymbol{\zeta}}) \leq \mathcal{Q}(\hat{\mu}_\mathrm{Y}, \bar{\Delta}_\mathrm{Y}, \bar{\Gamma}_\mathrm{Y}|\bar{\boldsymbol{\zeta}})$. Now to update Δ_Y, binary search finds a feasible $\hat{\Delta}_\mathrm{Y}$ on the line connecting $\bar{\Delta}_\mathrm{Y}$ and $\ddot{\Delta}_\mathrm{Y} = \operatorname{argmax}_{\Delta_\mathrm{Y}} \mathcal{Q}(\hat{\mu}_\mathrm{Y}, \Delta_\mathrm{Y}, \bar{\Gamma}_\mathrm{Y}|\bar{\boldsymbol{\zeta}})$. Thus

$\mathcal{Q}(\hat{\mu}_\mathrm{Y}, \bar{\Delta}_\mathrm{Y}, \bar{\Gamma}_\mathrm{Y}|\bar{\boldsymbol{\zeta}}) \le \mathcal{Q}(\hat{\mu}_\mathrm{Y}, \hat{\Delta}_\mathrm{Y}, \bar{\Gamma}_\mathrm{Y}|\bar{\boldsymbol{\zeta}})$. Finally, to update Γ_Y, binary search finds a feasible $\hat{\Gamma}_\mathrm{Y}$ on the line connecting $\bar{\Gamma}_\mathrm{Y}$ and $\ddot{\Gamma}_\mathrm{Y} = \mathrm{argmax}_{\Gamma_\mathrm{Y}} \mathcal{Q}(\hat{\mu}_\mathrm{Y}, \hat{\Delta}_\mathrm{Y}, \Gamma_\mathrm{Y}|\bar{\boldsymbol{\zeta}})$. Thus $\mathcal{Q}(\hat{\mu}_\mathrm{Y}, \hat{\Delta}_\mathrm{Y}, \bar{\Gamma}_\mathrm{Y}|\bar{\boldsymbol{\zeta}}) \le \mathcal{Q}(\hat{\mu}_\mathrm{Y}, \hat{\Delta}_\mathrm{Y}, \hat{\Gamma}_\mathrm{Y}|\bar{\boldsymbol{\zeta}})$. Thus, in summary, $\mathcal{Q}(\bar{\mu}_\mathrm{Y}, \bar{\Delta}_\mathrm{Y}, \bar{\Gamma}_\mathrm{Y}|\bar{\boldsymbol{\zeta}}) \le \mathcal{Q}(\hat{\mu}_\mathrm{Y}, \hat{\Delta}_\mathrm{Y}, \hat{\Gamma}_\mathrm{Y}|\bar{\boldsymbol{\zeta}})$.

C Mode of SN distribution

The mode of $\mathrm{SN}(\mu, \sigma, \lambda)$ is given by $\mu + \sigma m_0(\lambda)$, where $m_0(\lambda) = \sqrt{2/\pi}\delta - (1 - \pi/4)\frac{\left(\sqrt{2/\pi}\delta\right)^3}{1-(2/\pi)\delta^2} - \frac{\mathrm{sign}(\lambda)}{2}\exp(2\pi/|\lambda|)$

D Evaluating a pairwise density constraint

To evaluate a pairwise density constraint, say $f_\mathrm{SN}(\cdot;\theta_1) \succ f_\mathrm{SN}(\cdot;\theta_2)$, first we compare their modes. If $\mathrm{mode}(f_\mathrm{SN}(\cdot;\theta_1)) \le \mathrm{mode}(f_\mathrm{SN}(\cdot;\theta_2))$, then the constraint is not satisfied. If $\mathrm{mode}(f_\mathrm{SN}(\cdot;\theta_1)) > \mathrm{mode}(f_\mathrm{SN}(\cdot;\theta_2))$, then we test the other two conditions. For a fast comparison, we generate a set, $\mathbb{S}_\mathrm{grid}$, of 200 equally spaced points between the maximum and minimum of the top scores to limit the number of points for density comparison. Then we test $f_\mathrm{SN}(s;\theta_1) > f_\mathrm{SN}(s;\theta_2)$ ($f_\mathrm{SN}(s;\theta_1) < f_\mathrm{SN}(s;\theta_2)$) at all points $s \in \mathbb{S}_\mathrm{grid}$ that are above (below) $\mathrm{mode}(f_\mathrm{SN}(\cdot;\theta_1))$ ($\mathrm{mode}(f_\mathrm{SN}(\cdot;\theta_2))$). If it is true, we consider the contraint as satisfied, otherwise it is not satisfied.

E Binary search for density constraints

In Algorithm S1, the `densityContraintsSatisfied`$(\mathrm{Y}, \alpha_\mathrm{Y}, \bar{\boldsymbol{\zeta}})$ function is used to check if all pairwise constraints for component Y density would still be satified if one of its three parameters in $\bar{\boldsymbol{\zeta}}$ is updated to a new value α_Y. The pairwise density contraints are evaluated as described in Section D.

Algorithm S1 Binary search for density contraints.

Require: 1) $\mathrm{Y} \in \mathbf{Y}$: the component whose parameters are being updated, 2) $\ddot{\alpha}_\mathrm{Y} \in \left\{\ddot{\mu}_\mathrm{Y}, \ddot{\Delta}_\mathrm{Y}, \ddot{\Gamma}_\mathrm{Y}\right\}$: the new, possibly infeasible, estimate of one of the three SN parameters of Y from the parameter update equations in Section 4.3.2, and 3) $\bar{\boldsymbol{\zeta}}$: the current feasible estimate of all parameters. $\bar{\alpha}_\mathrm{Y}$, the current estimate of the same parameter as $\ddot{\alpha}_\mathrm{Y}$, is contained in $\bar{\boldsymbol{\zeta}}$.

Ensure: $\hat{\alpha}_\mathrm{Y}$: a feasible point on the line segment connecting $\bar{\alpha}_\mathrm{Y}$ to $\ddot{\alpha}_\mathrm{Y}$, as close to $\ddot{\alpha}_\mathrm{Y}$ as possible.

if `densityContraintsSatisfied`$\big(\mathrm{Y}, \ddot{\alpha}_\mathrm{Y}, \bar{\boldsymbol{\zeta}}\big)$ **then**
  ▷ All density constraints for component Y are satisfied when $\ddot{\alpha}_\mathrm{Y}$ replaces the corresponding parameter in $\bar{\boldsymbol{\zeta}}$.
  $\hat{\alpha}_\mathrm{Y} \leftarrow \ddot{\alpha}_\mathrm{Y}$
else
  while $\frac{|\ddot{\alpha}_\mathrm{Y} - \bar{\alpha}_\mathrm{Y}|}{\bar{\alpha}_\mathrm{Y}} > 10^{-4}$ **do**
    $\hat{\alpha}_\mathrm{Y} \leftarrow \frac{\bar{\alpha}_\mathrm{Y} + \ddot{\alpha}_\mathrm{Y}}{2}$
    if `densityContraintsSatisfied`$\big(\mathrm{Y}, \hat{\alpha}_\mathrm{Y}, \bar{\boldsymbol{\zeta}}\big)$ **then**
      $\bar{\alpha}_\mathrm{Y} \leftarrow \hat{\alpha}_\mathrm{Y}$
    else
      $\ddot{\alpha}_\mathrm{Y} \leftarrow \hat{\alpha}_\mathrm{Y}$
    end if
  end while
  $\hat{\alpha}_\mathrm{Y} \leftarrow \bar{\alpha}_\mathrm{Y}$
end if
return $\hat{\alpha}_\mathrm{Y}$

Note that if the current parameter, $\bar{\alpha}$, is feasible, the binary search is guaranteed to give a feasible solution as it can find a value arbitrarily close to $\bar{\alpha}$. Consequently, if the the first set of parameters are feasible, the approach is guaranteed to give a feasible solution at each iteration.

F One-sample Model Update Rules

Below are the update rules for the parameters of the component skew normal distribution.

$$
\begin{aligned}
w_{\mathrm{Y}} &= \frac{\sum_{s_1 \in \mathbb{S}_1} \bar{\omega}_{\mathrm{Y}}(s_1)}{|\mathbb{S}_1|} \\
\ddot{\mu}_{\mathrm{Y}} &= \frac{\sum_{s_1 \in \mathbb{S}_1} \bar{\omega}_{\mathrm{Y}}(s_1) \bar{m}_{\mathrm{Y}}(s_1, \bar{\Delta}_{\mathrm{Y}})}{\sum_{s_1 \in \mathbb{S}_1} \bar{\omega}_{\mathrm{Y}}(s_1)}, \\
\ddot{\Delta}_{\mathrm{Y}} &= \frac{\sum_{s_1 \in \mathbb{S}_1} \bar{\omega}_{\mathrm{Y}}(s_1) \bar{d}_{\mathrm{Y}}(s_1, \ddot{\mu}_{\mathrm{Y}})}{\sum_{s_1 \in \mathbb{S}_1} \bar{\omega}_{\mathrm{Y}}(s_1) \xi_2(s_1, \bar{\theta}_{\mathrm{Y}})}, \\
\ddot{\Gamma}_{\mathrm{Y}} &= \frac{\sum_{s_1 \in \mathbb{S}_1} \bar{\omega}_{\mathrm{Y}}(s_1) \bar{g}_{\mathrm{Y}}\left(s_1, \ddot{\mu}_{\mathrm{Y}}, \ddot{\Delta}_{\mathrm{Y}}\right)}{\sum_{s_1 \in \mathbb{S}_1} \bar{\omega}_{\mathrm{Y}}(s_1)},
\end{aligned}
$$

G Code Availability

https://github.com/shawn-peng/xlms

	Search time w/ decoys	Search time w/o decoys	Model run time	Target database size
ALott	35 h 13 m 10.0 s	20 h 22 m 12.0 s	6 m 40.7 s	4
Alinden	192 h 10 m 52.0 s	70 h 6 m 5.0 s	8 m 17.6 s	79
CPSF	2 h 0 m 4.0 s	37 m 5.3 s	6 m 5.2 s	28
D1810	18 m 34.4 s	10 m 9.3 s	3 m 49.8 s	10
MS2000225	21 m 1.4 s	7 m 19.7 s	4 m 6.6 s	5
QE	8 m 15.2 s	5 m 14.5 s	4 m 18.1 s	2
RPA	1 h 37 m 57.3 s	25 m 26.7 s	3 m 30.3 s	480
Alban	25 m 20.3 s	7 m 48.8 s	5 m 43.3 s	20
Ecoli	37 m 26.5 s	12 m 16.1 s	11 m 0.1 s	56
Peplib	3 m 41.9 s	2 m 15.3 s	4 m 3.4 s	9

Table S1: Comparison of running time for the database search and the 2SMix model. The 2SMix run time is reported as an average per restart. The experiments were exectued on AMD EPYC 7452 32-Core Processor @ 2.345 GHz with 251GB RAM for single thread time.

	self-links	cross-links	loop-links	mono-links	total	self-links ratio
ALott	7998	20500	659	3758	24917	0.32
Alinden	1277	2406	423	2647	5476	0.23
CPSF	414	633	126	804	1563	0.26
D1810	7	8	1	61	70	0.10
MS2000225	248	410	20	407	837	0.30
QE	0	0	0	1	1	0.00
RPA	0	16	9	57	82	0.00
Alban	84	197	21	142	360	0.23
Ecoli	112	168	28	617	813	0.14
Peplib	1662	1663	0	858	2521	0.66

Table S2: Counts of PSMs of each cross-linking type. Self-links are pairs of peptides that come from the same protein. Cross-links are pairs of peptides that come from the same protein or from different proteins. The total is the sum of cross-links, loop-links and mono-links. The self-links ratio is self-links/total.

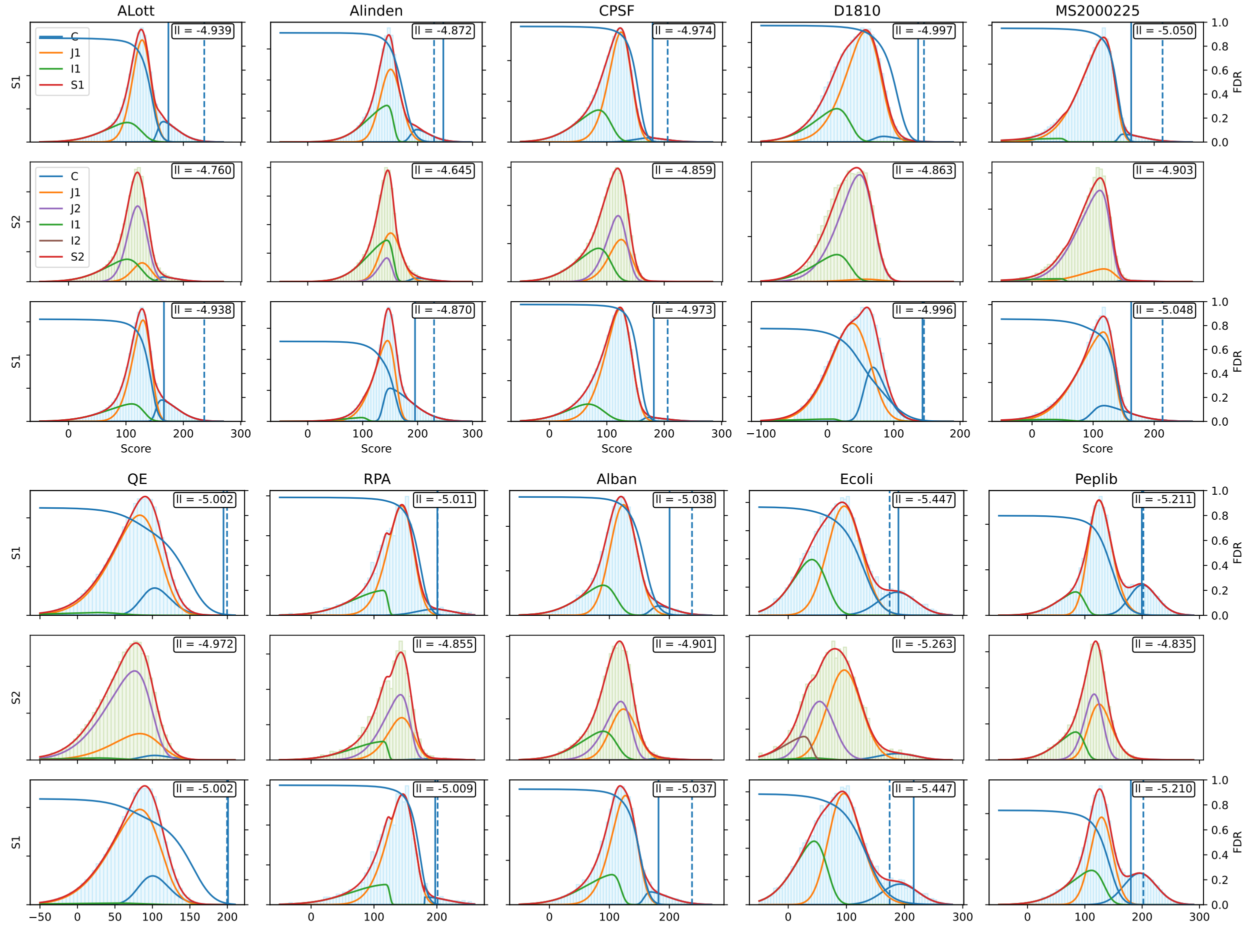

Figure S1: 1SMix and 2SMix model fits. 1SMix and 2SMix are the 1-sample and 2-sample mixture model respectively. The top two rows show the 2SMix fit on the top scores and the second scores. The third row shows 1SMix fit on the top scores. The empirical distributions of the top scores and the second scores are shown as histograms. The component densities estimated by the models are displayed. Each component density is weighted by its estimated mixture weight. The vertical solid line shows the 1% FDR threshold given by the mixture model, while the vertical dashed line shows the 1% FDR threshold by TDA for comparison. The average log-likelihood of the fitted model is shown in the upper right corner. For 2SMix the log-likelihood is computed separately for the top scores and the second scores.

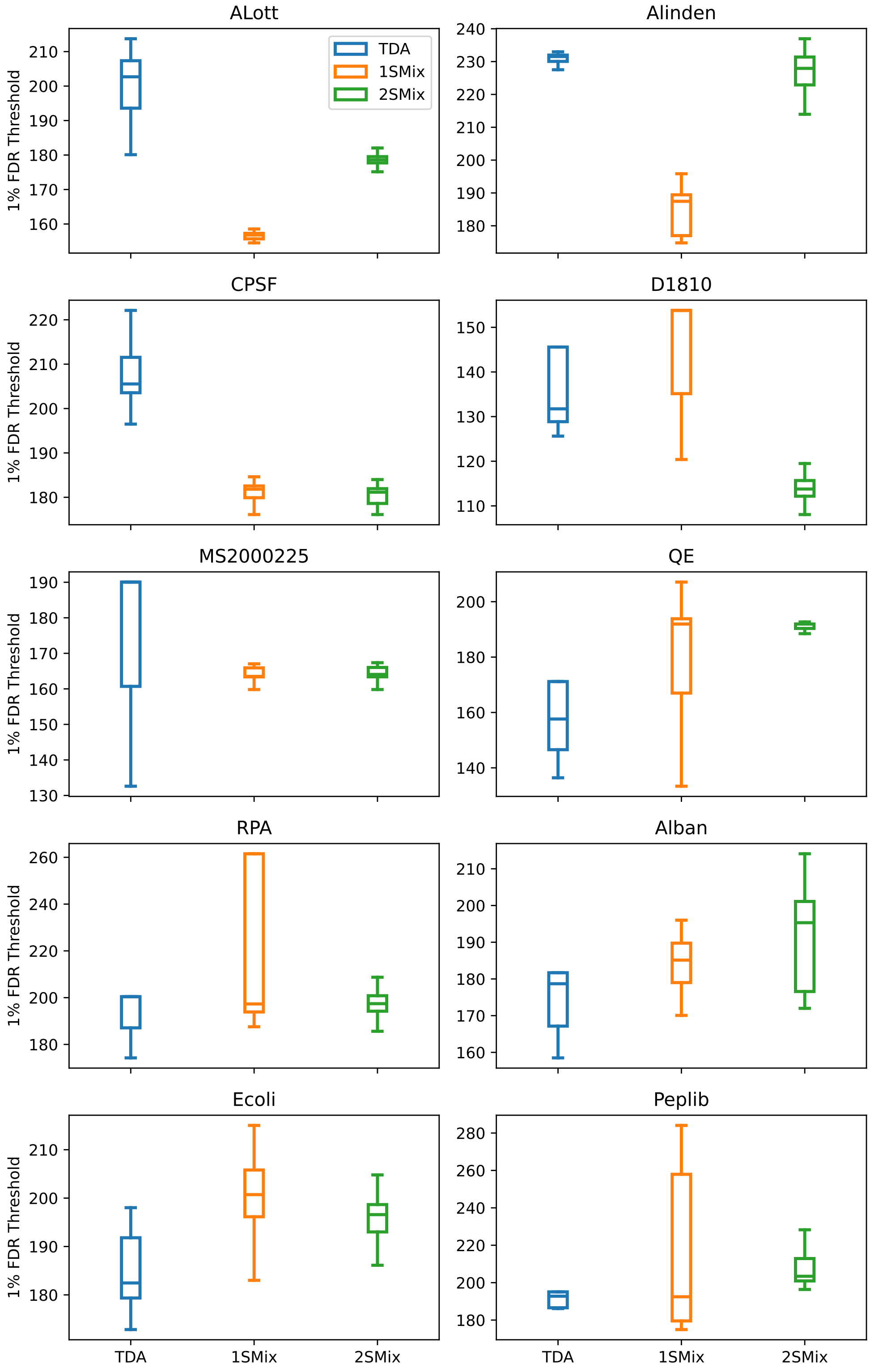

Figure S2: Stability of the 1% FDR threshold estimation. The stability of the methods was compared using 50 bootstrap samples, on which the 1% FDR thresholds were estimated. The larger spread of a boxplot indicates lower stability. Bootstrapping was performed on the set of original spectra.

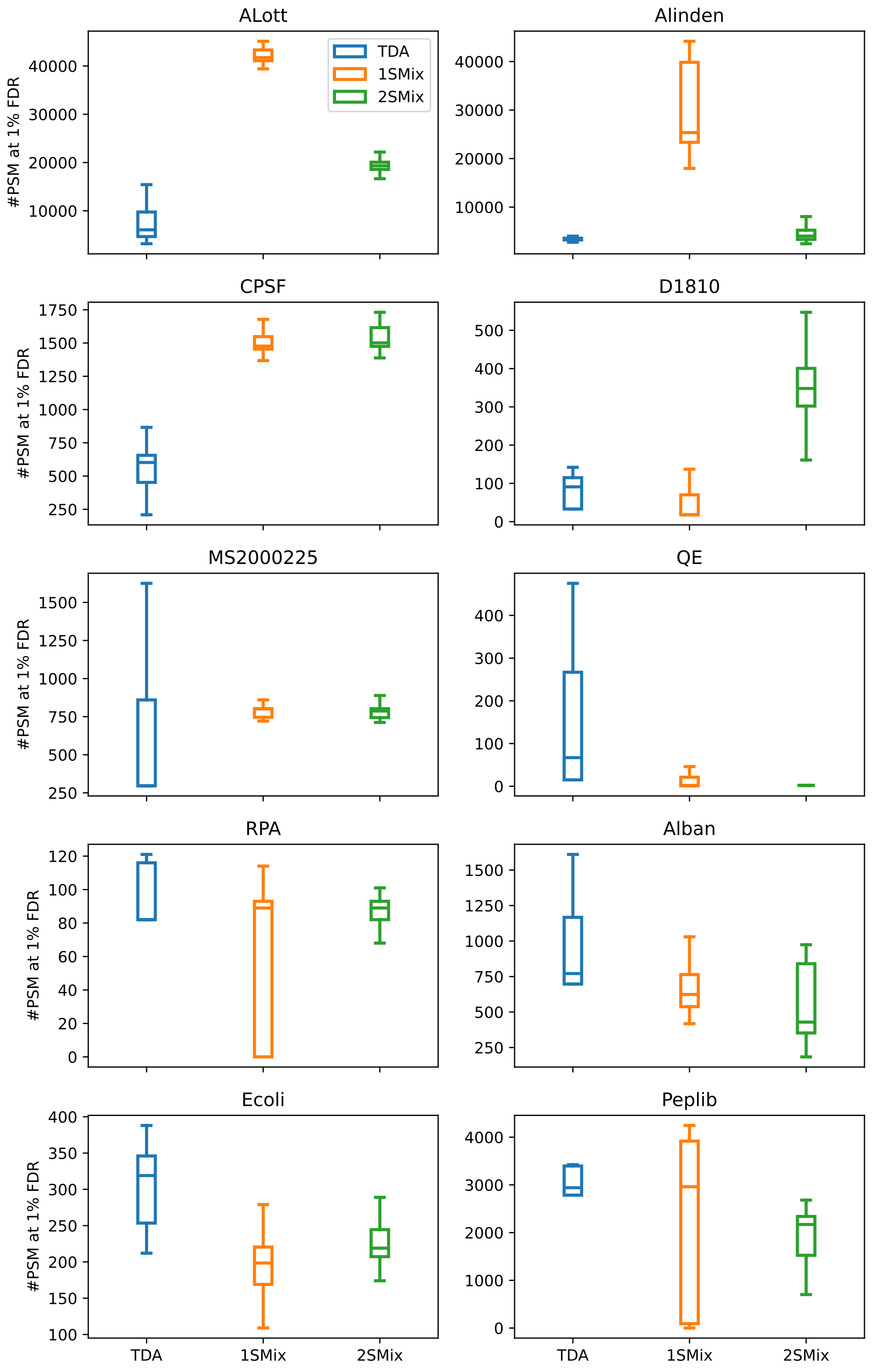

Figure S3: Stability of PSM identification. The stability of the methods was compared using 50 bootstrap samples, on which the number of PSMs scoring above 1% FDR threshold were counted. The larger spread of a boxplot indicates lower stability. Bootstrapping was performed on the set of original spectra.

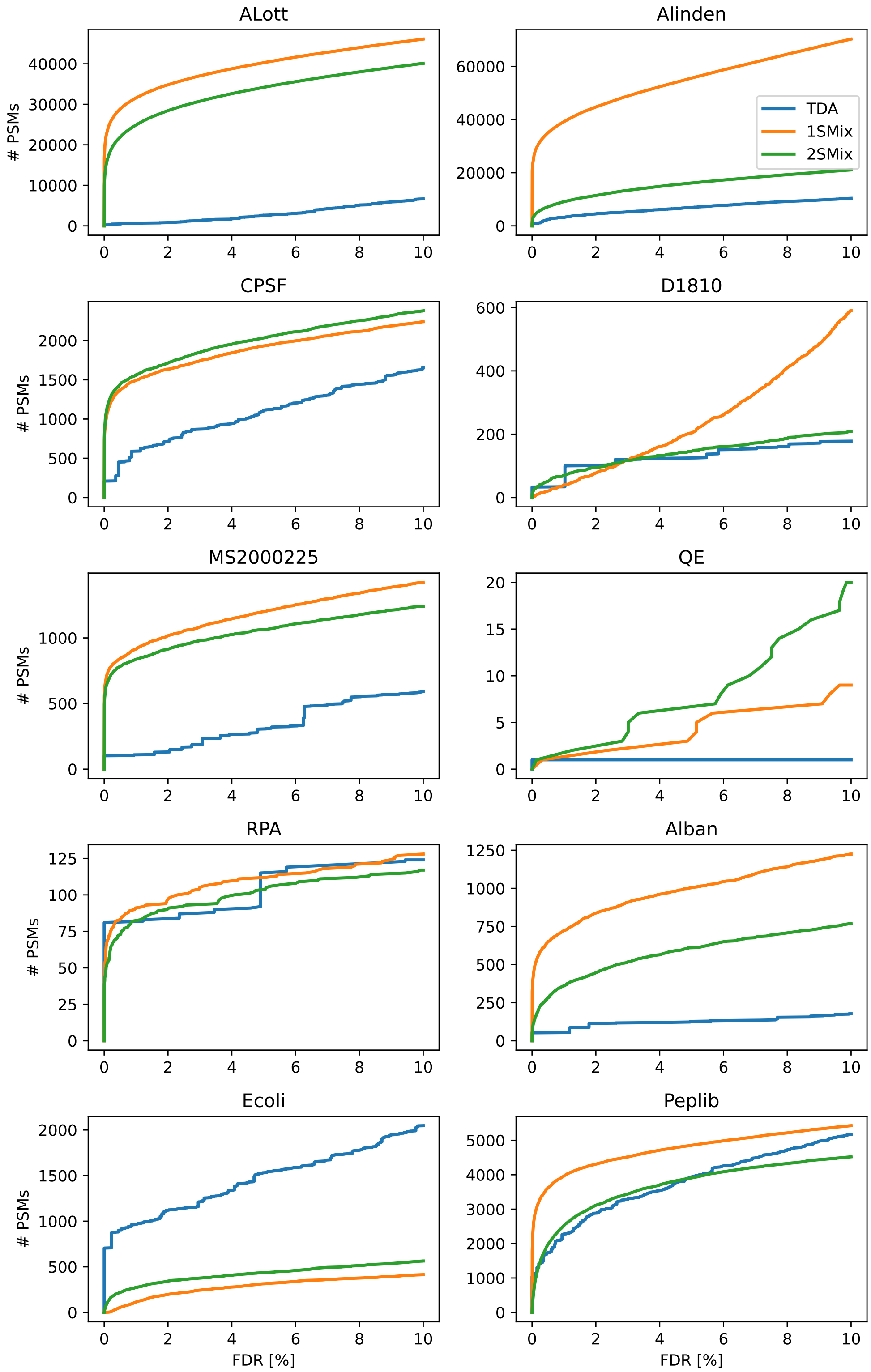

Figure S4: The number of identified PSMs as a function of FDR by 2SMix, 1SMix and TDA.

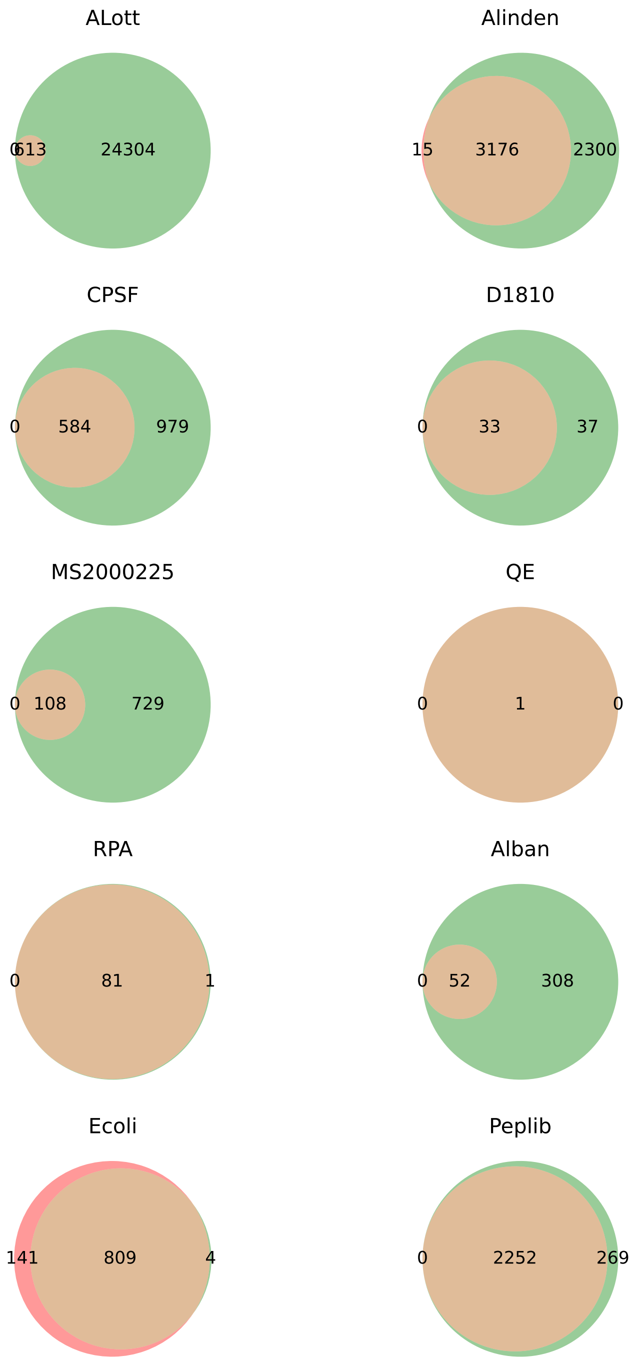

Figure S5: Comparison of PSMs identified by 2SMix and TDA at 1% FDR. PSMs identified by both 2SMix and TDA are in brown. Those identified by 2SMix, but not by TDA, are in green. Those identified by TDA, but not by 2SMix, are in pink.

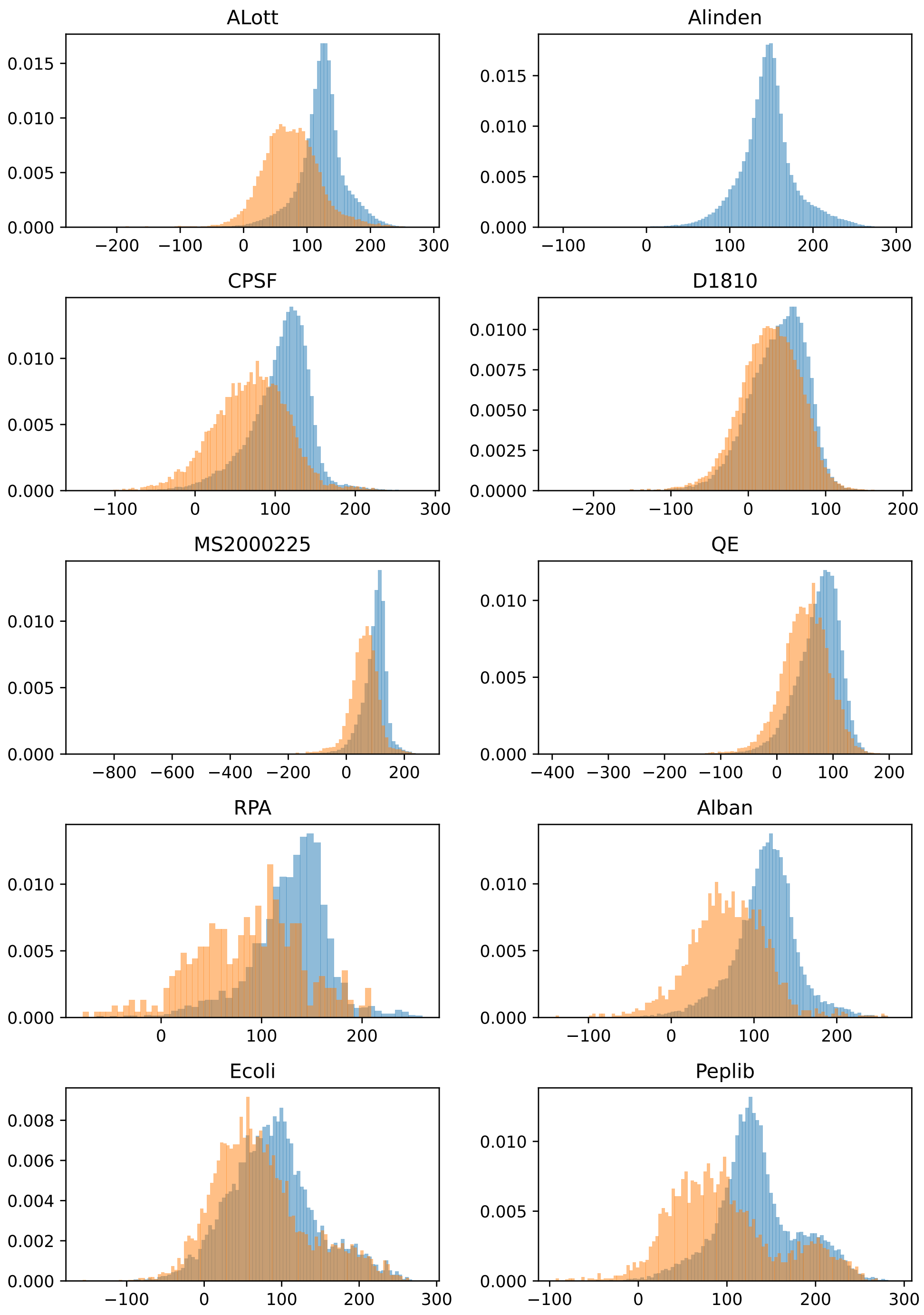

Figure S6: Second scores are not missing at random. The blue histogram is for the top scores from all spectra and the orange histogram is for the top scores whose spectra do not have a second hit. The second scores are not missing at random, since the top scores have a larger mass to the left.

References

[1] Mirjam Dür. Duality in global optimization: optimality conditions and algorithmical aspects. 1999.
