# Supplementary material for: An algorithm for decoy-free false discovery rate estimation in XL-MS/MS proteomics
Source: Bioinformatics. 2024 Jun 28;40(Suppl 1):i428–36. doi: 10.1093/bioinformatics/btae233 (PMC11256928; doi:10.1093/bioinformatics/btae233)
Supplement: btae233_Supplementary_Data [file Radivojac.246.response_to_reviews.pdf › btae233_Supplementary_Data/Radivojac.246.response_to_reviews.pdf]

Response to Reviews

Thank you for conditionally accepting our paper. We are pleased by the excellent quality of reviews, including the meta review, that identified both strong and weak points of our paper. We respond to all reviewers' comments below and believe that this work is suitable for publication at ISMB 2024.

SUBMISSION: 246
TITLE: An algorithm for decoy-free false discovery rate estimation in XL-MS/MS

------------------------ METAREVIEW ------------------------
Your manuscript has been thoroughly reviewed by at least three reviewers. Below is a summary of some main strengths and weaknesses identified by the reviewers.

Strengths:
- The method does not rely on so-called decoy models, i.e. explicit null models
- Use an expectation maximization-based framework for assessing the parameters of a mixture model of "skewed normal" distributions
- Multiple constraints are enforced on both mixture weights and density functions, to retain structural relationships between the top 2 scores
- The resulting model succeeds at achieving 4-fold running time reduction, increases in inference stability and reaches a similar accuracy in comparison to decoy-based approaches
- A novel approach to assess peptide identification reliability in the context of crosslinking mass spectrometry.

Thank you. We agree that these are the strengths of our paper.

Weaknesses:
* The mixture model seems fundamentally flawed: sharing the same skew normal components between the top score (S1) and the second best score (S1) is wrong. Please address this concern from Reviewer 3 to explain why the theoretical foundation of the mixture model is still reasonable.

All models of real-world phenomena are limited (flawed) owing to their assumptions and so we believe that "fundamentally flawed" is a strong language here. It could similarly apply to TDA. For example, the preprint by He et al. mentioned by Reviewer #3 states (in abstract) about TDA "We prove that… [TDA] provides a conservative estimate of the FDR for any given score threshold, but cannot rigorously control the FDR" and "We prove that the spectrum level FDR control is difficult". Given that, we think our model is useful at the very least to provide independent assessment whether TDA gives a meaningful solution on a particular dataset.

If we understand correctly, the reviewer might have drawn that conclusion based on the observation that the scores from I_1, J_1 and C in S_1 are likely to have higher values than those in S_2 on average due to the competition between the components. In the strict sense, the reviewer is correct, although we believe our model is a very good approximation. A more precise modeling would require a consideration of S_1 and S_2 with a max operation; e.g., S_1 = max{C, I_1, J_1}, instead of pure mixtures. It would further require capturing the conditional dependence between S_1 and S_2 at each score s. The error of modeling that and the difficulty of finding optima with such a difficult objective may combine to exceed the error made by our current model. We are very much aware of these issues and plan to derive more sophisticated approaches in future work.

Theoretically, the strength of our approach is that the skew normal distributions are excellent for modeling competition (e.g., competition of incorrect PSMs will lead to the I_1 score distribution). There exists a proof (now cited in the revised work) that the maximum or minimum between two Gaussian variables with the same parameters has a skew normal distribution (right or left skewed, respectively), and empirically that the maximum over several such variables looks similar (no parametric form is known to us). Therefore, these mixing (latent) distributions are a solid choice to model competition between PSMs. Another strength of our approach is that it uses S_2 score distribution to better resolve mixing components. This in fact is key to our method because otherwise, it would suffer from the same

problem that PeptideProphet does in traditional LC-MS/MS, as mentioned by Reviewer #2. Finally, our approach performs constrained optimization and imposes meaningful criteria on the weights and mixing components, thus further guiding the solution in the right direction. We believe, and it is empirically supported by our experiments, that the solutions found through this optimization are pretty good.

We encourage the (meta)reviewers to compare this formal albeit limited DFA approach with what is currently used as the FDR formula in TDA (Section 2.3)

FDR = (#TD - #DD) / #TT

For example, if there are more Decoy-Decoy (#DD) hits than Target-Decoy (#TD) hits, the FDR will be negative. But if there are many Target-Decoy hits, then adding some Decoy-Decoy hits will actually reduce FDR. While the formula is good in the steady state with large numbers, we think that it is problematic for low values of FDR, exactly where it's supposed to be used. Unfortunately, these problems are very hard to empirically demonstrate using currently available data.

* Numerous results should be explored with further depth. For instance, the authors do not show if they identify the same crosslinks as the TDA approach.

We agree that more empirical work would support our case and have added several results in the Supplement.

First, we added Venn diagrams; see Figure S5 in the Supplement. Note that the ranking of the identified PSMs is always the same (with a small discrepancy that the ranking of peptides with I vs. L can sometimes be permuted and cause minor discrepancies), causing that one set of identifications is generally a proper subset of the other, depending which method finds a lower FDR score threshold.

Second, we have added variability of the number of identified PSMs due to bootstrapping, as requested by the reviewer; see Figure S3.

Third, we have also added a report of the self-links in each mixture, see Table S2 in the Supplement.

Fourth, we timed the database searches and also found the run time for our method (Table S1).

Finally, we could not add the results for multiple search engines. This was certainly our initial plan, however, some other search engines are either not available any longer (XQuest) or do not allow us to extract the score of the second-best PSM (Kojak, XLSearch). We therefore had to limit our approach to a single search engine where we were able to extract the score of the second-best PSM for each spectrum.

* The performances against a target-decoy strategy to estimate false discovery rates are improved in some datasets but not in others. The authors do not show that the improvements in performances are legitimate. The authors should add some experiments to test the methods on some test datasets where certain crosslinks are known such as self-crosslinks to show that their results are correct.

Thank you. We now report the numbers of different types of peptides (or peptide pairs), including those within the same protein (self-links), across two different proteins (proper cross-links), loop-links, and mono-links.

The improvement in the performance is difficult to estimate because in MS/MS there is no ground truth and even a potential use of synthetic libraries can lead to inaccuracies due to errors in peptide synthesis and competition for ionization (see Bohrer et al., 2010, Analytical Chemistry). In the case of XL-MS/MS, we are not aware of a single such dataset and have therefore limited our work to showing that DFA achieves similar results but without extra search of the decoys.

Please do your best to revise your manuscript to address the weaknesses above as well as other specific comments made by the reviewers in their individual reviews. The weaknesses marked by "*" above are the concerns that must be addressed before the final acceptance of your manuscript.

Thank you. We have done so below.

Also, please make sure the software tool and dataset of your method are available at GitHub or other public code/data repositories for the community to use. This is required by the final acceptance of a manuscript.

The GitHub repository link was available in the Supplement of our paper. It is now also shown in the Abstract of the main manuscript.

The changes made on the manuscript need to be highlighted (e.g., in red font or yellow background). A response letter describing your point-to-point response to each concern above must be provided.

We have highlighted all non-trivial changes. A number of spelling and minor grammar mistakes were fixed without highlighting to not distract the reader from the main points. We moved the section Pairwise density constraints next to the section Component parameter updates to improve the flow. We now define the Lagrangian objective in section Weight updates under constraints and rename Section 4.4.1 as Derivation of the Q-function. Several new theoretical results are added in the Supplement since the paper itself cannot be extended beyond 9 pages.

----------------------- REVIEW 1 ---------------------
SUBMISSION: 246
TITLE: An algorithm for decoy-free false discovery rate estimation in XL-MS/MS
AUTHORS: Yisu Peng, Shantanu Jain and Predrag Radivojac

----------- Overall evaluation -----------
Peng et al. describes a method for assessing confidence for peptide-spectrum matches from cross-linkeíng mass spectrometry. The method's main advantage is that it does not rely on so-called decoy models, i.e. explicit null models. Instead, the authors use an expectation maximization-based framework for assessing the parameters of a mixture model of "skewed normal" distributions.

The algorithm is well described, particularly it is interesting to learn how to use constraints between the mixture models' parameters, without any violations of the EM algorithm's assumptions.

Thank you.

I only have a minor comment. In Fig 1 the AUthors plot several series that should be better described. It seems like "IC" is what previously been described as "J". Also, there are several blue lines, where of not all could be the "C" series.

Thank you. We have improved the quality of the plots to be suitable for the publication. We did notice the IC vs. J problem shortly after submitting this work and have fixed it in the revised version.

----------------------- REVIEW 2 ---------------------
SUBMISSION: 246
TITLE: An algorithm for decoy-free false discovery rate estimation in XL-MS/MS
AUTHORS: Yisu Peng, Shantanu Jain and Predrag Radivojac

----------- Overall evaluation -----------
The manuscript introduces a non-decoy algorithm for FDR estimation cross linking mass spectrometry, based on two-sample mixtures of skew-normal distributions. The proposed method is a generalization of the one published by the authors for conventional LC-MS/MS data in 2020. Multiple constraints are enforced on both mixture weights and density functions, to retain structural relationships between the top 2 scores.

A collection of ten datasets from PRIDE from vastly different organisms including H.sapiens, bacteria (E.Coli), plants (A. thaliana), and fungi (C. thermophilum) were used for method evaluation. The resulting model succeeds at achieving 4-fold running time reduction, increases in inference stability and reaches a similar accuracy in comparison to decoy-based approaches.

Thank you. We agree with your assessment.

The authors use a massive amount of maths to make sure that the mixture distribution actually does something reasonable. From our own experience, we can say that fitting some parameterized distribution to an empirical score distribution can very easily go berserk. That is one of the reason why Bayesian methods such as PeptideProphet were almost completely replaced by TDA when it was developed. But the authors do a rather convincing job on the 10 datasets to show how good their fitting works in practice.

Thank you. Yes, we agree with your statement about PeptideProphet and there are nice studies describing the exact phenomenon (cited in our work). We believe that one of the main reasons why PeptideProphet fails in difficult cases is that it is a one-sample approach; that is, it relies only on the scores of the highest scoring PSM for each experimental spectrum. An equivalent approach in our paper (also in the previous Peng et al. 2020 paper) does not do too well either in some cases. The score distribution of the second-best scores is critical to properly resolving the mixing components.

Some minor details could be improved such as:

* Do not use abbreviations in the title

The title would be overly long if we used “cross-linking tandem mass spectrometry” instead of XL-MS/MS. Instead we have added “proteomics” at the end of the title to help the reader.

* Some writing mistakes could be fixed (e.g Section 5.1 "..the fit is excellent is all cases...")

Thank you. We have fixed multiple typos and reworded sentences that weren’t clear. Truth be told, there were embarrassingly many typos in the submitted manuscript. We apologize for that. It turned out that the spell checking in our Overleaf project was accidentally turned off which caused us to believe the manuscript was clean at the submission stage. We have taken care of it now.

* Lack of description/instructions in GitHub repo.

Thank you. We have been adding text to the GitHub repo.

----------------------- REVIEW 3 ---------------------
SUBMISSION: 246
TITLE: An algorithm for decoy-free false discovery rate estimation in XL-MS/MS
AUTHORS: Yisu Peng, Shantanu Jain and Predrag Radivojac

----------- Overall evaluation -----------
ISMB 2024 Submission 246
An algorithm for decoy-free false discovery rate estimation in XL-MS/MS

0: borderline paper

The paper introduces a new, decoy free approach (DFA) for estimating/controlling the FDR in cross-linking tandem mass spectrometry proteomics. The core of the new method is based on a 2020 paper by a group of authors that includes the current three authors and which uses a mixture of skew normal distributions to model the score of the top 1 or the top 2 peptide-spectrum match (PSM) scores.

The paper is mostly well written and the analysis is interesting and fairly sophisticated. However, as explained below, I am afraid I have some significant reservations about this work.

Thank you.

First, the mixture model seems fundamentally flawed: sharing the same skew normal components between the top score (S1) and the second best score (S1) is wrong because, for example, when conditioned on the correct score being the overall second best score (S2), its distribution should be different than if we know it's the overall top score (S1). I believe the authors are aware of this issue, based on a comment they made discussing the ALott dataset in Results, but I feel this is a much more serious problem than something that can be brushed aside as a comment in Results.

The reviewer is correct in that the model is not accounting for conditional dependencies between the scores for the first and second hit, as discussed above. Indeed, we are aware of this issue, but unfortunately, a model that does incorporate these additional dependencies is quite complex. The approach we took is as far as we could push it within a couple of years of work. Having said that, we believe that the model we have, though approximate, has its advantages over TDA and therefore is worth considering.

Second, I am not sure that this work represents progress over the commonly practiced target-decoy approach (TDA). Specifically, my issue is that TDA is supported by rigorous theoretical results (He et al., 2015). Granted, these theoretical results rely on assumptions whose validity might be disputed, but at least we have a solid theoretical baseline. After carefully reading this paper it isn't clear to me if there is a corresponding theoretical basis for the proposed approach.

We have discussed the theoretical basis for this model above in the comments to the meta reviews. We believe this method has a solid grounding, despite the limitations in modeling conditional distributions. The He et al. (2015) work does provide a theoretical basis for TDA, but it certainly highlights its limitations in rigorously estimating FDR as well. We have also now cited He et al. (2015).

Rather, it seems to me that the proposed approach is empirically driven, and as such I would find it more attractive if there is some very convincing evidence that it has a clear advantage over TDA. I'm afraid I can't really see such evidence in the current manuscript. For starters, the authors claim their method offers "significant run-time savings" but there isn't a single benchmark to support this claim. Moreover, while in some cases their method delivers significantly more discoveries than TDA does, there is no convincing argument why we should trust that they properly control the FDR in those cases.

The time savings are in the search, which is based on a single database instead of a concatenated database.

On this latter point I argue that even if the fit between the estimated mixture distribution and the empirical histogram is excellent, it does not follow that the components and their weights are accurately estimated. This is important because it is the latter rather than the overall fit that the FDR estimates rely on. I find this criticism particularly relevant in light of the first issue I raised about the flawed model.

The reviewer is correct in that the overall fit is not as important as getting the latent distributions right. In our previous work (Peng et al, 2020), there were existing datasets that could be used to check the validity of modeling and FDR estimation. However, with cross-linking MS/MS, these datasets do not exist and one cannot verify either TDA or DFA, unless one can interpret a large number of spectra manually. We have used here TDA results and interpreted our ability to mostly match the estimated FDR as a good outcome for our work, and an empirical validation of the assumptions. However, more work is needed and datasets where ground truth is known. It may be necessary to develop synthetic datasets for future purposes.

Some other comments I have:

- There are a few clear typos ("upto", "the the", "appraoch", etc.) which should be easily fixed with a spellcheck.

Thank you for noticing those. As described above, spelling typos were all fixed, as well as some grammar issues here and there.

- Section 3 is not detailed enough: how exactly are the target and decoy paired databases constructed?

We have slightly modified the language in the manuscript. We have used the reversed protein sequences as decoy.

- "Missing second scores occur preferentially at the left and right tails of top score distribution" - while I can see why the second score would be missing for low scores, it's not clear to me why it would be missing for high scores.

Thank you for asking this question. Our initial statement was somewhat ad hoc, based on several examples we observed. Based on your question, we now plot the histogram (Figure S6) of top scores when the second score is missing and indeed they preferentially fall on the left side.

- I believe you are using ECM rather than EM, and as such I am not sure your claim that "In each iteration our EM algorithm finds \xi that maximizes" is correct. Specifically, I doubt that the 3-step maximization process you apply (4.4.2) is equivalent to finding the overall maximum with respect to the k-step parameter values.

The reviewer is correct, we are indeed using the ECM algorithm. The update equations do not find the local maximum of the Q-function. We optimize a single parameter at a time by taking a derivative of the Q-function with respect to it, keeping other parameters constant, equating it to 0, and solving the resulting equation. Though a local maximum of the Q-function is not obtained, the new parameters increase the value of the Q-function, thereby also increasing the log-likelihood as per the ECM theory [1]. We will modify the main text by referring to our algorithm as an ECM based approach, including appropriate references. And expand on the derivation of our update equations in the Supplement.

[1] Meng, Xiao-Li, and Donald B. Rubin. "Maximum likelihood estimation via the ECM algorithm: A general framework." Biometrika 80.2 (1993): 267-278.

- Section 4.4.4: please clarify if the same heuristic you apply to \mu is also applied to \Delta and \Gamma. Also, can you comment on the effect these adjustments might have on EM's critical property of the increasing likelihood?

Yes, the same heuristic is indeed applied to \Delta and \Gamma. The increasing likelihood of EM and ECM algorithms relies on finding a set of parameters that increase (precisely, do not decrease) the Q-function. This property is still satisfied by the parameters obtained from the binary search. As a function of a single parameter, keeping other parameters fixed, the Q-function is unimodal, i.e., has a unique stationary point giving the global maximum and has no local minimum. For \mu and \Delta the unimodality is a consequence of the concavity of the Q-function w.r.t. to those parameters individually and the existence of a stationary point. In the case of \Gamma, though the Q-function is not concave, we show that it is still unimodal. Now, each parameter update in Section 4.4.2 finds the new parameter as the local maximum of the Q-function, which due to the unimodality is the same as the mode. Since the binary search is performed between the old and the new parameter, the estimated parameter, on the line connecting them, is guaranteed to not decrease the value of the Q-function. We have included a formal proof of this property in the Supplementary Materials.

We also want to point out that due to the inequality constraints the updated weight parameters are not guaranteed to maintain the monotonicity of the Q-function and the log-likelihood in each iteration. However, experimentally we still observe the log-likelihood to increase over multiple iterations.

- Figure 1: is IC in the legend the same as J from the text? Is the decreasing curve the estimated FDR?

Yes, the IC was our original internal notation that we replaced in the manuscript with J for brevity. We have now fixed those issues. The decreasing blue curve is indeed the estimated FDR. We have added language in the legend to describe it as indeed it was originally missing.

- 5.1: I wouldn't consider yours and TDA's FDR thresholds for Alinden and D1810 as "nearly identical".

This is a fair point. We have changed the language to “similar”.

- "yet some of the highly scored second hits cannot be correct identification" - please clarify.

This was concluded by manual inspection of the PSMs and looking at the matching peaks.

- Figure 2: showing the variation in the number of discoveries might be more informative.

Thank you. We now also report the variability in the number of discoveries at 1% FDR. These results are in the Supplementary Materials now.

- "they should have higher mean values than incorrect partially incorrect matches" - missing an "or"?

Thank you. We have added “or” here.

- 5.2: please provide details on how the bootstrap experiments are done.

Bootstrapping was done on the initial set of spectra in each dataset. These spectra were bootstrapped and the procedure was identical thereafter. We have updated the paper to clarify this.

- 5.3: talks about identified peptides but refers to Figure 3, which discusses identified PSMs - please clarify.

Thank you. We changed “peptides” into “PSMs”

- "However, the field is confronted with computational and statistical challenges (Aggarwal et al., 2022)" - is a paper on issues in estimating the FDR in proteogenomics really proving your point here?

We have removed this reference, thank you.

- "We extensively evaluated this method to show that the low-variance high-quality FDR estimation can be achieved without requiring decoy data" - I have to disagree with you here on whether you established this "high-quality FDR estimation".

Strictly speaking you are correct; however, in this case, no one has established that TDA is high-quality either. We have addressed this issue by removing high, thus leaving “quality FDR estimation”. We think it’s an acceptable compromise.

----------------------- REVIEW 4 ---------------------
SUBMISSION: 246
TITLE: An algorithm for decoy-free false discovery rate estimation in XL-MS/MS
AUTHORS: Yisu Peng, Shantanu Jain and Predrag Radivojac

----------- Overall evaluation -----------
The manuscript entitled "An algorithm for decoy-free false discovery rate estimation in XL-MS/MS" presents a novel approach to assess peptide identification reliability in the context of crosslinking mass spectrometry. The authors compare their approach against a method based on a target decoy database search using 10 datasets.

Overall Evaluation:
The authors did an excellent job at providing background introduction on the topic. The methods are well described for the most part. However, the results are under explored and under analyzed.

Thank you. We have generated additional results requested by the reviewers. Most of those will be placed in the supplement.

Comments that must be addressed:
Numerous results should be explored with further depth. For instance, the authors do not show if they identify the same crosslinks as the TDA approach. Simple Venn diagrams would do the trick for this.

Thank you. We have now included those in the Supplementary Materials (Figure S5)

Also, the performances against a target-decoy strategy to estimate false discovery rates are improved in some datasets but not in others. The authors do not show that the improvements in performances are legitimate. The authors should select datasets where certain crosslinks are known such as self-crosslinks to show that their results are correct.

The reviewer is correct about that, but unfortunately, the ground truth in MS/MS data is not known and usually it is difficult to definitively prove an advantage in accuracy.

The TDA approach used in this study is extremely simplistic and relies on a single database search score. Such approach is extremely uncommon in today's world and most TDA approaches rely on multiple scores and pieces of data to score peptide spectrum matches and estimate FDR. A modern TDA-based FDR estimation method should be used as benchmark.

We believe that however the original TDA may be modified, at the end there will be a single ranking of peptide pairs for each experimental spectrum, the best of which will be processed further. Therefore, our approach would be equally applicable to alternative TDA approaches. We are not entirely sure what procedure the reviewer had in mind, and will be happy to answer this question more concretely if the question was more specific.

The code should be made available in a public repository such as GitHub.

The code is available. The link was provided in the Supplement, but we have moved it to the main manuscript now.

It is unclear how the greedy algorithm picks which constraints to apply when selecting two. Is it attempted for all pairs of constraints? This should be clarified as it can have a significant impact on the results.

When updating a component's single parameter (\mu or \Delta or \Gamma), we check all pairwise density constraints where the component is involved. The binary search is performed to satisfy all pairwise constraints involving the component being updated.

In case of the weight constraints, we first check if a feasible solution can be obtained without explicitly enforcing any inequality constraint. If some inequality constraints are violated, we solve the system of equations with one violated inequality constraint as active at a time. The order of picking the active constraint is determined based on the index of the constraint. For violated inequality constraints i and j, i is applied as active before j if i < j. If a feasible solution is not obtained with the single active constraints, a set of two active constraints are applied simultaneously. Each pair of violated constraints is identified as a pair of sorted indices, i.e. constraints 4 and 3 are represented as (3,4). Each pair of violated constraints is applied in the lexicographical ordering of the sorted index based representation; i.e., (3,4) is applied before (3,5) and (4,2). The same lexicographical ordering is used when considering 3 or more inequality constraints simultaneously.

We have added these details in the Supplementary Materials.

The database search parameters are not described in the Methods.

Thank you. All database search parameters are listed in Table 1.

The TDA results are missing from the QE dataset in Figure S3.

The curve was missing because the number of identified PSMs for TDA was 0. We have now fixed this problem. The results are now in Figure S4.

Section 2 and 3 should be in the Methods section.

We appreciate the advice, but we actually prefer the layout we originally chose.

The manuscript contains a fair number of typos. Page 6 contains a typo: "As per the KKT theory, the soultion," and should read: "As per the KKT theory, the solution,". The same applies for "inequality constriants", which should read "inequality constraints".

The reviewer is correct. We have fixed all typos, as mentioned above. We did not notice the spell checker was turned off which led us to believe everything was clean.

In Page 8 Section 5.3 "Figure 3 shows the number of identified peptieds as a function" should read "Figure 3 shows the number of identified peptides as a function". In the same section, "resutls" should be spelled "results"

Thank you, fixed.

Figure 1's x-axis is not labeled.

Thank you. We have now labeled all axes in all Figures.

Suggestions:
Most methods that estimate false discovery rates in the context mass spectrometry-based proteomics data analysis will show their methods performance irrespective of the database search algorithm used. This is not done here.

As mentioned above in response to the meta review, there are four search engines for XL-MS we are aware of: XQuest, Kojak, XLSearch and OpenPepXL. To our surprise, XLQuest was not available any longer for download, while Kojak and XLSearch do not allow extraction of the second-best score. We were therefore left with a single search engine for our approach.
